# Biodiversity assessment among two Nebraska prairies: a comparison between traditional and phylogenetic diversity indices

**DOI:** 10.3897/BDJ.3.e5403

**Published:** 2015-07-17

**Authors:** Shelly K. Aust, Dakota L. Ahrendsen, P. Roxanne Kellar

**Affiliations:** ‡ University of Nebraska at Omaha, Omaha, United States of America

**Keywords:** asterids, community ecology, conservation, grasslands, next-generation sequencing, phylogenetic diversity

## Abstract

**Background:**

Conservation of the evolutionary diversity among organisms should be included in the selection of priority regions for preservation of Earth’s biodiversity. Traditionally, biodiversity has been determined from an assessment of species richness (*S*), abundance, evenness, rarity, etc. of organisms but not from variation in species’ evolutionary histories. Phylogenetic diversity (PD) measures evolutionary differences between taxa in a community and is gaining acceptance as a biodiversity assessment tool. However, with the increase in the number of ways to calculate PD, end-users and decision-makers are left wondering how metrics compare and what data are needed to calculate various metrics.

**New information:**

In this study, we used massively parallel sequencing to generate over 65,000 DNA characters from three cellular compartments for over 60 species in the asterid clade of flowering plants. We estimated asterid phylogenies from character datasets of varying nucleotide quantities, and then assessed the effect of varying character datasets on resulting PD metric values. We also compared multiple PD metrics with traditional diversity indices (including *S*) among two endangered grassland prairies in Nebraska (U.S.A.). Our results revealed that PD metrics varied based on the quantity of genes used to infer the phylogenies; therefore, when comparing PD metrics between sites, it is vital to use comparable datasets. Additionally, various PD metrics and traditional diversity indices characterize biodiversity differently and should be chosen depending on the research question. Our study provides empirical results that reveal the value of measuring PD when considering sites for conservation, and it highlights the usefulness of using PD metrics in combination with other diversity indices when studying community assembly and ecosystem functioning. Ours is just one example of the types of investigations that need to be conducted across the tree of life and across varying ecosystems in order to build a database of phylogenetic diversity assessments that lead to a pool of results upon which a guide through the plethora of PD metrics may be prepared for use by ecologists and conservation planners.

## Introduction

Preservation of Earth’s biodiversity is a priority as ecosystems face changes due to anthropogenic actions, which initiate rapid adaptive responses from organisms, affect genetic variation (often depleting it) in extant species, and result in the establishment of new communities (Santamaría and Méndez 2012). Conservation of biodiversity leads to stable communities which provide ecosystem services for humans (e.g. water purification, erosion control, climate regulation) (Balvanera et al. 2006, Santamaría and Méndez 2012). There is a modern movement to preserve evolutionary diversity among species, but selections of priority regions for conservation have traditionally been based on species richness and diversity.

Since MacArthur (1965) seminal paper on species diversity, species richness (*S*), the count of all species in a sample, has been one of the most commonly used indices for selecting conservation areas (i.e. higher species richness = greater biodiversity; e.g. Pavoine and Bonsall 2011, Gotelli and Chao 2013, Van Meerbeek et al. 2014). Beyond simple species counts, two widely recognized similarity indices – Jaccard Index (*S*_J_; Jaccard 1912) and Sørensen Index (*S*_S_; Sorensen 1948) – have been used to distinguish biodiversity content between geographic sites using species’ presence/absence data (Gotelli and Chao 2013). *S*_J_ is the amount of homogeneity or shared diversity between sites and compares the number of shared species to the total number of species in the combined communities (Gotelli and Chao 2013). *S*_S_ applies weight to species that are common to each site over those found at only one site by comparing the number of shared species to the mean number of species in a single community (Gotelli and Chao 2013). More recently, scientists and stakeholders have called for clearer and more rigorous means of characterizing biodiversity value, such as with phylogenetic approaches (Rolland et al. 2011).

Biodiversity assessment should start with both knowledge of the species present and their evolutionary histories (Steele and Pires 2011). Phylogenetic diversity (PD) indices account for evolutionary differences between species in a community (Forest et al. 2007, Winter et al. 2013). Selecting geographic regions with greatest PD for protection will conserve the greatest diversity of organismal features upon which evolutionary forces may act; therefore, preservation of PD is acknowledged as the best way to maintain effective ecosystems (Forest et al. 2007). Since the introduction of the original PD metric (PD_Faith_; Faith 1992), many additional metrics have been developed based either on species presence/absence data or abundance data. Incorporating abundance into PD metrics may allow ecologists to better understand how evolutionary history impacts ecosystem processes and provides a method of comparing PD with traditional diversity indices (Cadotte et al. 2010a).

Some of the most common PD metrics are shown in Table 1, and they assimilate branch length data differently. The way that each metric is calculated (i.e. summation of branch lengths, diverse averages of branch lengths, etc.) determines the aspect of biodiversity highlighted. For example, some metrics emphasize phylogenetic relationships deep in the tree (e.g. NRI, MPD, and PSV; Webb 2000, Helmus et al. 2007) and others draw attention to relationships near the tips of the tree (e.g. NTI, MNTD, and PSC; Webb 2000, Helmus et al. 2007). A review of the formulas used to calculate each metric is beyond the scope of this article and multiple extensive reviews of various PD metrics have already been conducted (for example, see Vellend et al. 2011, Winter et al. 2013, Pearse et al. 2014, Kellar et al. 2015a). However, see “Discussion” below for varying interpretations of the metrics calculated in this study. Previous empirical studies have compared a few of these metrics, but none have conducted a broad investigation that allows for direct comparison between all of the common metrics based on the same dataset.

Until recently, most studies in which PD was examined used simulated data or only one to a few gene sequences downloaded from GenBank (e.g. Webb 2000, Helmus et al. 2007, Cadotte et al. 2012). These sequences include the two most commonly available plastid protein-coding genes for plants – *rbcL* (Chase et al. 2005) and *matK* (Chase et al. 1993, Johnson and Soltis 1994, Cadotte et al. 2009). Another common method to produce phylogenies is to attach taxa without branch lengths to a megatree. These trees provide low resolution below the family level (Srivastava et al. 2012) and may rely on node-based metrics which are not as useful as metrics based on topology and branch length (Winter et al. 2013). Some studies indicate that polytomies (unresolved relationships) in a phylogeny result in uninformative values of PD metrics that use branch lengths (Srivastava et al. 2012, Van Meerbeek et al. 2014), while others suggest polytomies have little effect on detecting correlations between PD and ecological patterns at higher taxonomic levels (Cadotte et al. 2008, Cadotte et al. 2009, Flynn et al. 2011). While previous studies agree that incorporating PD into evolutionary, ecological, and conservation investigations is important, more empirical studies are needed that address the effect of phylogenetic resolution on PD metrics and compare PD with traditional diversity indices. Our study allows for this comparison and a discussion about how some PD metrics can be used to describe the phylogenetic structure of a community. The increasing availability of phylogenetic information and methods to incorporate them into investigations is also providing a framework for understanding community assembly.

Individuals in a community interact based on the traits they possess. Traits can be traced through evolutionary history; therefore, phylogenies can give an indication of how members of a community assemble (Webb et al. 2002). Over evolutionary time, the presence of high trait variation due to phylogenetic diversity increases above-ground productivity and is associated with greater ecosystem and community stability due to the utilization of unshared resources or facilitative interactions (Cadotte et al. 2012). A community consisting of closely related species is phylogenetically clustered (i.e. low diversity), while a community consisting of distantly related species is phylogenetically overdispersed or evenly dispersed (i.e. high diversity). As environmental conditions change, a phylogenetically overdispersed community has a better chance to adapt and maintain community and ecosystem functioning (Cadotte et al. 2010a). Another means of assessing community assembly is through assessment of functional diversity, the third primary component of biodiversity (the other two being species diversity and phylogenetic diversity as described by Swenson 2011).

Functional diversity (FD) evaluations highlight complementary or differing patterns of community assembly that influence biodiversity and community function. Phylogenetic diversity and FD assessments are good indicators of the effects of biodiversity on ecosystem function (Cadotte et al. 2011, Flynn et al. 2011); however, they may be only weakly correlated (Cadotte et al. 2011, Flynn et al. 2011). Studies have shown that ecosystem function may be predicted from PD assessments (Cadotte et al. 2008, Flynn et al. 2011), but the exact underlying mechanisms are not well understood (Srivastava et al. 2012). It is not known which functional traits are best represented by PD (Flynn et al. 2011), and because high quality trait data for many species is lacking (Flynn et al. 2011), PD can be used to summarize multiple traits into a simple index when multiple plant traits cannot be measured (Cadotte et al. 2008, Flynn et al. 2011, Srivastava et al. 2012). Comparing FD and PD may allow for predictions about how species will respond to environmental changes over time and how those changes will affect ecosystem services (Díaz et al. 2013).

In this study, we utilized massively parallel (also known as next-generation) sequencing to generate DNA character data from three cellular compartments (plastids, mitochondria, and nuclei) in plants. These data were used to estimate both robust, total evidence phylogenies with high bootstrap support and single- and dual-gene phylogenies in order to test the effect of data quantity on PD metrics. With these phylogenies, we calculated and compared 17 PD metrics, four traditional diversity indices, and the phylogenetic signal of one plant functional trait among plants in two Nebraska prairies. Our study aimed to answer the following questions: 1) How do datasets of varying character quantities affect PD metrics? and 2) What do the various metrics indicate about biodiversity at these sites?

## Methods

*Study sites* – Our research focused on two endangered prairies in Nebraska, U.S.A.: 1) The Nature Conservancy’s Niobrara Valley Preserve (NVP; 23,000 hectares) located in north-central Nebraska (42^o^47' N, 100^o^02' W) and 2) Nine-Mile Prairie (NMP; 93 hectares) located northwest of Lincoln, Nebraska (40^o^52' N, 96^o^48' W). These sites were selected because remnant prairies have decreased in total geographic area more than any other ecosystem since the early 1800s (Samson and Knopf 1994), and yet, prairies are among the most biologically productive of all ecosystems (Williams and Diebel 1996). Understanding biological diversity in prairies is vital to protecting the few remaining relicts. These two sites are similar in that they have never been plowed, but they differ in abiotic conditions such as soil composition, allowing for a diversity comparison with few variables other than variation in species content. Additionally, biodiversity assessments at these sites provide a basic framework of data upon which future comparisons across the latitudinal diversity gradient and between varying ecosystems may be made. In addition to calculating metrics for NVP as a whole, we compared three distinct sites within NVP (North, South, and West). Field work covered approximately 2,100 hectares at NVP (North = 270 ha; South = 1060 ha; West = 832 ha) and the entire 93 hectares at NMP.

*Taxon sampling* – Ideally, a biodiversity study should assess all organisms in a community; however, this is not practical due to time and financial limitations. Grasses make up most of the biomass in prairies, but flowering forbs (i.e. herbaceous non-grasses) make up the greatest diversity (Turner and Knapp 1996), and studies have shown that the diversity of plants in a community strongly influences the diversity of other organisms (e.g. arthropods; Dinnage et al. 2012). To involve as many closely-related flowering forb species as possible such that DNA sequences would align cleanly for phylogenetic analyses, we focused on asterids, which include many of the dominant forbs in prairies and are found all over the world with over 80,000 species (Bremer et al. 2004). Additionally, asterids make up approximately 33% of angiosperm species in prairies (based on species lists for NMP and NVP as well as other prairies in North America). Taxon sampling included species from multiple families across the asterid clade as well as samples from Santalales and Caryophyllales, plant orders outside the asterid clade (Chase and Reveal 2009) that were included as outgroups.

Field work was conducted in 2012 and 2013. Three samples of each asterid or outgroup species found at the sites were collected for herbarium vouchers, and fresh leaf material was collected and dried over silica for DNA extractions. Rare species and small populations (i.e. less than 20 individuals) were not collected in order to protect the species’ populations. Using a field sub-sampling of random 1m x 2m plots, we estimated the total *S* at each site with a species accumulation curve. We located plots at all points at which a ‘new’ species occurred plus multiple plots selected at random to ensure full coverage of the sites. We recorded plot locations on a Trimble GPS and mapped them in Arc/GIS. Maximum *S* was identified when the accumulation of additional asterid and outgroup species ceased to increase regardless of the number of additional plots examined. For each plot, we recorded the percent cover (abundance) of each species. All species were identified by morphological characters using *The Flora of Nebraska* (Kaul et al. 2011), and all collections were deposited in OMA and NEB herbaria.

*DNA extraction and sequencing* – Total genomic DNA including plastid (cp), mitochondrial (mt), and nuclear (nr) DNA was extracted using the IBI Genomic DNA Mini Kit (IBI Scientific, Peosta, IA, USA) until 12 μg of DNA, measured with a NanoDrop (Thermo Scientific), was obtained. Samples were sent to the University of Nebraska Medical Center or University of Missouri DNA Core for library preparation and Illumina sequencing. Samples were run on Illumina Hi-Seq at 14 samples per lane, paired-end, or 12 samples per lane, single-pass runs. In addition to several new species collected and sequenced for this study, we included 76 cp genes from 23 Asteraceae species published in Kellar et al. (2015b)​.

Illumina sequence reads were mapped to a reference genome (from the same family or a close relative) downloaded from GenBank (Benson et al. 2005). Reads were mapped to complete plastid and mitochondrial genomes, and the 18S, 5.8S, and 26S nrDNA sequences in Geneious 6.1.7 (Biomatters, Inc., www.geneious.com) using referenced-based mapping, medium sensitivity, up to five iterations. We also pulled mt genes and nrDNA regions from the previously sequenced Asteraceae species (Kellar et al. 2015a). Genes and nrDNA harvested from the consensus sequences were concatenated and aligned using the MAFFT (Katoh et al. 2002) plug-in in Geneious, and alignments were uploaded to the Dryad Digital Repository (http://dx.doi.org/10.5061/dryad.qj177; Aust et al. 2015​). To address the question of how differing datasets affect PD metrics, four datasets were assembled: 1) *rbcL* only, 2) *matK* only, 3) *rbcL*+*matK*, and 4) cpmtnuc (concatenation of all plastid, mitochondrial, and nuclear regions).

*Phylogenetic analyses* – Phylogenetic analyses were conducted with both maximum parsimony (using PAUP* 4.0b10; Swofford 2002) and maximum likelihood (ML; using Garli 0.951; Zwickl 2006) algorithms. Garli was accessed through the CIPRES Science Gateway on-line portal (Miller et al. 2010). Maximum parsimony bootstrap analyses were performed on 1,000 replicates with one random addition per replicate. Modeltest (Posada and Crandall 1998) was used to determine the model of evolution for ML analyses, resulting in the selection of the GTR + I + G model. Maximum likelihood bootstrap analyses were performed on 1,000 replicates using an automated stopping criterion set to 20,000 generations.

*Metric calculations* – To compare *S* between sites, we calculated the effective number of species (ENS) by taking the exponential of the Shannon-Wiener index (a non-linear index), which accounts for the entropy in a set of samples (Jost 2006). ENS reveals the number of equally common species and is called the “true diversity” by Jost (2006). We converted the Shannon-Wiener index to ENS such that the diversity between sites could be assessed. Additionally, we calculated *S*_J _(Jaccard 1912) and *S*_S _(Sorensen 1948) to assess similarity between sites. These the traditional diversity indices were compared to *I*_ST_, a similarity index that incorporates phylogeny (described below). ENS, *S*_J_, and *S*_S_ (Table 1) were calculated using the software program EstimateS (Version 9; Colwell 2013).

All PD metrics were calculated in R (Version 3.0.1; R 2013) using the Picante package (Kembel et al. 2010) and the R function listed in Table 1. We did not use rate-smoothed trees as PD estimates show only minor influences from subtle branch length transformations (Cadotte et al. 2008, Cadotte et al. 2009). To assess the statistical significance of the resulting values, each PD metric was compared to a null distribution generated from 10,000 randomizations of the phylogeny. Parametric statistical tests cannot be used to compare the various PD metrics between sites because each metric produces a single data point for each site. However, some of the metrics were used to rank sites from low to high diversity, and then a non-parametric rank-based statistic was calculated to compare NMP to NVP (Mann-Whitney statistic) as well as compare the three sites within NVP (Kruskal-Wallis statistic).

To provide one example of how assessment of functional diversity may be incorporated into this type of study, we measured the phylogenetic signal of specific leaf area (SLA; leaf area:dry mass). SLA indicates the amount of matter a leaf invests in order to produce energy via photosynthesis (Cornelissen et al. 2003, Dwyer et al. 2014). Studies have found that SLA varies between plants in a population, as well as between leaves on the same plant (Poorter and De Jong 1999, Dwyer et al. 2014). In order to generate an average SLA for each species, we collected three fully mature leaves with petioles intact and free from damage from three separate plants (a total of nine leaves). Fresh leaves were placed beside a metric ruler, flattened by a piece of clear plastic, and images were taken with a digital camera. Leaves were then placed in coin envelopes and were dried over silica. Captured images of fresh leaf material were loaded into Image J (Girish and Vijayalakshmi 2004) to measure leaf area. Dried leaves were weighed to obtain dry mass.

To quantify the phylogenetic signal of this functional trait, SLA was mapped on the phylogeny by assigning the SLA value to the corresponding tree tip (the corresponding extant species). The *K* statistic (Blomberg et al. 2003) was calculated using the Picante package (Kembel et al. 2010) in R statistical software (Version 3.0.1; R 2013). The *K* statistic reveals the likelihood that phylogenetically related species resemble each other in a trait across a tree. The measured value indicates trait convergence (*K*<1; i.e. species resemble each other less than expected by chance), trait conservatism (*K*≥1; i.e. species resemble each other more than expected by chance), or that a trait changed at a small constant rate under the Brownian motion model (*K*=1; Baraloto et al. 2012). To assess statistical significance, each resulting *K* value was compared to 1,000 randomizations of the phylogeny.

## Data resources

The data underpinning the analysis reported in this paper are deposited in the Dryad Data Repository at http://dx.doi.org/10.5061/dryad.qj177.

## Results

DNA extractions for 40 collections (see Suppl. material 1 for herbarium accession numbers) were sent for Illumina sequencing. These samples were chosen based on their quality (i.e. they had the highest DNA yield). For each species, we recovered 76 plastid genes, six mitochondrial genes, and three nrDNA regions (see Suppl. material 2 for lists of genes/regions included and GenBank accession numbers). The genes were chosen based on recoverability, meaning they had adequate Illumina read coverage (Straub et al. 2012) in most of the species. Coverage of plastid assemblies ranged from 101x to 5113x (mean = 840x), mitochondrial assemblies from 8x to 11385x (mean = 547x), and nrDNA assemblies from 1781x to 12294x (mean = 1781x). In addition to the 63 samples that we processed [Suppl. material 1; 40 sequenced here plus 23 from Kellar et al. (2015b)​], we downloaded cpDNA, mtDNA, and nrDNA from GenBank for two additional species: Asteraceae *Helianthus annuus* (GenBank accession numbers: cp: NC_007977; mt: NC_023337, nr: KF767534) and Apocynaceae *Asclepias syriaca* (GenBank accession numbers: cp: NC_022432; mt: NC_022796; nr: JF312046). All phylogenetic analyses were based on a total of 65 species (62 asterids and three outgroups).

**Figure 1.**
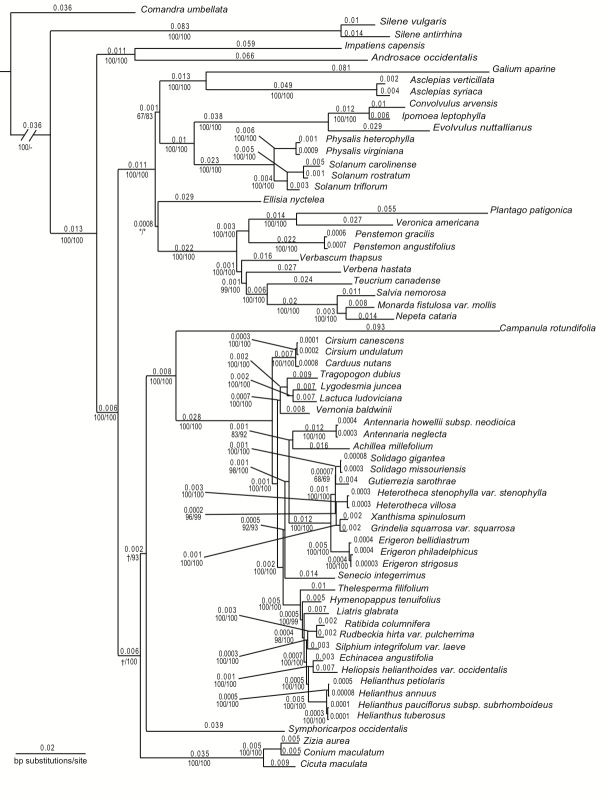
Maximum likelihood (ML) tree (-ln L=46268.63) inferred from the concatenation of 76 plastid, six mitochondrial, and three nuclear ribosomal repeat regions (cpmtnuc; Suppl. material 6​); matching the single most parsimonious (MP) tree except were dagger (†) is shown. Tree includes 62 asterid species and 3 outgroups (*Comandra umbellata*, *Silene vulgaris*, and *Silene antirrhina*). Numbers above branches indicate branch lengths used to calculate various Phylogenetic Diversity (PD) metrics. Numbers below the branches indicate MP/ML bootstrap support values resulting from 1000 replicates each. Low branch support (<50) is indicated by an asterisk (*). Missing bootstrap values are denoted by a dash (-).

Phylogenetic trees were estimated: 1) *rbcL* only (Suppl. material 3), 2) *matK* only (Suppl. material 4), 3) *rbcL* + *matK* (Suppl. material 5), and 4) cpmtnuc (Fig. 1), and tree statistics were assembled (Table 2). The tree inferred from *rbcL* only (Suppl. material 3) contained many branches with weak (<50) bootstrap support. Bootstrap support improved in the *matK* and *rbcL* + *matK* trees (Suppl. materials 4, 5), but these trees also included relationships with weak support. The cpmtnuc tree (Fig. 1) had the best bootstrap support overall [i.e. most branches had strong (>85) bootstrap scores]; however, this tree contained one branch with weak support. For all datasets, the ML tree was congruent with one of the maximum parsimony trees, except where noted. Branch lengths from the ML trees were used in all PD metric calculations because ML results in a single tree that has the highest probability of giving rise to the observed data.

Four traditional diversity indices and 17 PD metrics were calculated using the cpmtnuc tree (Table 3) for the two prairies (NMP and NVP) and for the three sub-sites within NVP (North, South, and West). Metric values that were statistically different from random are marked with an asterisk. The abundance-weighted metric values (those indicated with “aw” subscript in Table 3) were often less than their non-abundance-weighted counterparts. Correlations (regressions not shown) between species- incidence and abundance-weighted metric values were mixed (MPD and MPD_aw_: r^2^ = – 0.135; MNTD and MNTD_aw_: r^2^ = – 0.765; NRI and NRI_aw_: r^2^ = – 0.097; NTI and NTI_aw_: r^2^ = 0.611; SPD and SPD_aw__: _r^2^ = 0.836). Note that *S* S, *S* J, and *I* ST are pairwise comparisons between NMP and NVP, between the three sites within NVP, and between NMP and each of the three sites at NVP. *S* J, *S* S, and *I* ST assessed biotic similarity and differences between sites, and regressions were calculated for each pair of indices as follows: *S* J and *S* S: r^2^ = 0.941, *S* J and *I* ST and *S* S and *I* ST: r^2^ = – 0.758 for both.

We conducted regression analyses (not shown) to estimate the relationships between *S* and several PD metrics. A strong positive correlation was seen between *S* and PD_Faith_ (r^2 ^= 0.974), a moderate positive correlation between *S* and MPD (r^2 ^= 0.562), a weak negative correlation between *S* and MNTD (r^2 ^= – 0.110), and a strong positive correlation between *S* and SPD (r^2 ^= 0.975). In addition, comparisons between *S* and PSV (r^2 ^= 0.058) and between *S* and PSE (r^2 ^= 0.016) revealed no correlation, *S* and PSC (r^2 ^= 0.4885) showed a weak correlation, and *S* and PSR (r^2 ^= 0.984) showed a strong positive correlation.

**Figure 2.**
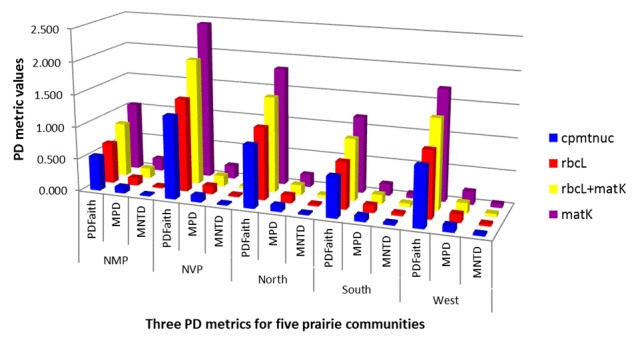
Comparison of three PD metrics (PD_Faith_, MPD, and MNTD) calculated from varying datasets: *rbcL, matK, rbcL* + *matK*, and cpmtnuc for five prairie communities. Notes: cpmtnuc = concatenation of 76 plastid genes, six mitochondrial genes, and three nuclear repeat regions; NMP = Nine-Mile Prairie, NVP = Niobrara Valley Preserve, and North, South, and West represent the three sites within NVP

To address the question of how datasets containing different amounts of data affect PD metrics, the three most common metrics (PD_Faith_, MPD, and MNTD) were compared (Fig. 2). With few exceptions, metric values for all communities were lowest when calculated from the cpmtnuc tree and highest when calculated from the *matK* tree. Of these values, only MNTD calculated from the single- and dual-gene phylogenies for NMP and the MNTD value from the dual-gene phylogeny for West were statistically significant. The remaining values were not significantly different from random. We conducted regression analyses (not shown) to assess the correlation between *S* and each metric calculated from the four different datasets. Relationships were consistent across the varying datasets as follows (average r^2^): strong correlation between *S* and PD_Faith_ (r^2 ^= 0.95); moderate positive correlation between *S* and MPD (r^2 ^= 0.38); and a weak negative correlation between *S* and MNTD (r^2 ^= – 0.03).

The phylogenetic structure of each community can be revealed by several of the PD metrics (PD_SES_, NRI, NRI_aw_, NTI, NTI_aw_). However, most of the metric values in this study were not statistically significant, and in these cases, the results suggest random assembly. Only one value was statistically significant (NTI for NMP) indicating the species were phylogenetically clustered at this site.

Results of the non-parametric rank-based comparison (ranks not shown) revealed that NMP tended to rank lower in diversity than NVP across the metrics (*U*_1 _= 6.84; *P* = 0.009). In addition, the South community tended to rank lower in diversity than the North or West communities (F_2 _= 2.03; *P* = 0.362), although this result was not statistically significant.

SLA was calculated for each species, and average values ranged from 17.5 to 773.9 cm^2^/g (Suppl. material 1). Once the SLA trait was mapped onto the tree, the *K* statistic was calculated (Table 3). Only South had a *K* statistic greater than one, indicating phylogenetic clustering of this functional trait.

## Discussion

Conservation biologists, community ecologists, and other researchers are currently exploring new ways to compare and contrast biodiversity between communities and ecosystems. With the growing popularity of massively parallel DNA sequencing and the ease of estimating or availability of existing phylogenies, these researchers are exploring phylogenetic diversity metrics. However, with the plethora of PD metrics now available, researchers are seeking advice as to which PD metrics should or may be used in various situations (Winter et al. 2013). This guidance will best be provided by comparing the various PD metrics between communities based on a common dataset. In this investigation, we calculated 17 PD metrics and compared them to four traditional diversity metrics and one example of a functional diversity trait among two endangered prairies in Nebraska, U.S.A. Additionally, we compared a few of the most common PD metrics calculated from a multi-gene (cpmtnuc) phylogeny to those calculated from single- (*rbcL* or *matK*) or dual-gene (*rbcL*+*matK*) phylogenies to determine the effect of varying quantities of data on PD metrics. Below we discuss the specific questions addressed in this study.

*How do datasets of varying character quantities affect PD metrics?* – The three most common PD metrics (PD_Faith_, MPD, and MNTD) were calculated based on four datasets varying in DNA character (nucleotide) quantity (Fig. 2). The single-gene datasets (*rbcL* and *matK*; Table 2) had few nucleotide differences between species, resulting in poor phylogenetic resolution and poor bootstrap support for many clades (Suppl. materials 3, 4). This is despite the fact that these two genes are the most common markers in plant systematics (Chase et al. 2005). The dual- (*rbcL* + *matK*; Suppl. material 5) and multi-gene (cpmtnuc; Fig. 1) trees had more differences, and therefore, better resolution and stronger branch support for clades. Additionally, the resulting PD metric values for each community were lower when calculated from the cpmtnuc tree than PD metrics calculated from single- and dual-gene trees (Fig. 2). This is not surprising because branch lengths are measured in average number of nucleotide substitutions per site. Therefore, because many coding regions have very few nucleotide differences between taxa, the longer the sequence alignment, the lower the *average* number of substitutions per site. However, the phylogenies estimated from many genes had better resolution and greater bootstrap support for relationships because the *total quantity* of nucleotide substitutions increased with an increased number of genes (see “# Parsimony informative characters” in Table 2).

We cannot compare the absolute values of these PD metrics from varying datasets because of the differences in how the branch lengths are measured; therefore, to determine if they are characterizing biodiversity differently, we analyzed the change in each metric across the species gradient at the different sites (see regression values in “Results”). The correlations were the same despite the difference in character data used to calculate the PD metrics; however, some correlations were as expected from simulations (Cadotte et al. 2010a, Tucker and Cadotte 2013), but others were not. Computer modeling has shown a strong positive correlation between *S* and PD_Faith_ when the species pool contains less than 80 taxa and no correlation between *S* and MPD (Tucker and Cadotte 2013). Our data showed these correlations because our species pools were all less than 80, but our data did not match the predicted relationship between *S* and MNTD. Modeling has shown a strong negative correlation between these variables, but our data showed only weak negative correlation (average regression for all datasets, r^2 ^= – 0.03). This difference may indicate a non-random change in species relatedness as *S* changes or may be the result of small sample size.

These results suggest that a multi-gene phylogeny may not be necessary to obtain relevant PD metric results; however, one must proceed with caution. First, our results highlight the importance of using comparable datasets (i.e. the same character matrix) when inferring phylogenies to calculate and compare PD metrics between sites because of the incorporation of branch lengths. Supertrees constructed from smaller phylogenies that were likely estimated from different datasets cannot be used to calculate PD metrics. Second, this is the first study to address this question with a large clade of flowering plants, but the sample size is relatively small. Additional studies are needed that make these same calculations with larger datasets across varying communities/ecosystems.

*What do the various metrics indicate about biodiversity at these sites?* – Scientists from multiple fields of study seek comprehensive biodiversity assessment tools and empirical studies that reveal proper application of the multitude of metrics. Phylogenetic, functional, and species diversity are the main components contributing to biodiversity (Swenson 2011), and our study highlights the value of incorporating all three components into diversity investigations. Here we review and compare multiple diversity metrics.

Global conservation organizations select priority regions for preservation based on several factors, but they have all considered *S* as a basic index for characterizing biodiversity (e.g. Myers 1988, Olson and Dinerstein 2002). To get an idea of diversity beyond simple species counts, the easiest index to calculate is ENS. When all species in the community are equally abundant, ENS should equal *S.* When the value of ENS for a community is higher than *S* it means there is more diversity than expected, and when ENS is less than *S*, diversity is lower than expected. This index can be used to compare the diversity between two communities with equal numbers of species. For example, in our study, for South and NMP, *S* was the same (22 species), but ENS was different (ENS_South_ = 56.9; ENS_NMP_ = 31.6), revealing greater diversity in South than NMP. When communities have differing *S* values, ENS does not necessarily indicate higher or lower diversity relative to each other. Metrics that directly compare similarities and differences between sites include *S*_J_, *S*_S_, and *I*_ST_.

*S*_J_ and *S*_S_ measure site similarities and do not include phylogeny, whereas *I*_ST_ measures site differences and incorporates phylogenetic information; therefore, *S*_J_ and *S*_S_ are expected to be positively correlated, and *S*_J_ and *I*_ST_ and *S*_S_ and *I*_ST_ are expected to be negatively correlated. Our data matched these expectations, providing multiple lines of support for the site comparison metrics. Beyond the traditional diversity measures, conservation organizations may want to select priority regions based on evolutionary history of species but may not have the resources to assemble phylogenetic information. Therefore, it is important to know if and when *S* can be used as a predictor of phylogenetic diversity.

It may seem obvious that a tree with more species will have more branches and a high probability of having greater PD_Faith_ (Calba et al. 2014). This relationship was confirmed by computer modeling studies of Tucker and Cadotte (2013), but the strong positive relationship between *S* and PD_Faith_ was limited to datasets containing less than 80 species. This may explain the correlation across our five sites in which *S* ranged from 22 to 55 species. However, there was one exception in our data. *S* was equal at NMP and South, but South had a higher value of PD_Faith_, indicating the 22 species at South are more evolutionarily distinct (have higher diversity) than the 22 species at NMP. Our result indicates that *S* may or may not be a good predictor of PD_Faith _(i.e. overall phylogenetic diversity) when species pools are small. However, comparing values of PD_Faith _directly between communities can reveal those that have increased evolutionary potential (those with higher PD_Faith_; Forest et al. 2007).

Our empirical data resulted in mixed correlations between *S*, SPD, and the Helmus et al. (2007) PD metrics. Helmus et al. (2007) reported no correlation between *S* and PSE but found a correlation between *S* and PSR, and these predictions matched our results. Likewise, our data showed a strong correlation between *S* and SPD. Because PSR and SPD both incorporate *S* into their products and PSR characterizes biodiversity similarly to PD_Faith_ (Helmus et al. 2007), these results are not surprising. Helmus et al. (2007) also predicted a correlation between *S* and PSV, but our data showed no correlation, perhaps because our results for PSV were all in the middle of the potential 0–1 range of values (Table 3). These mixed results are likely due to a small *S*, but they provide a basis upon which future investigations may expand and lead to stronger conclusions about how these metrics perform on different datasets.

Mean pairwise distance (MPD) averages the evolutionary differences between all pairwise species in the tree and reveals deep species relatedness. Higher values indicate more species with above-average branch lengths. Mean nearest taxon distance (MNTD) averages the evolutionary distance between each species and its nearest neighbor. Higher values indicate that some taxa have branches that are much longer than average. Net relatedness index (NRI) and nearest taxon index (NTI) are equivalent to MPD and MNTD, respectively, but they compare MPD and MNTD values to null communities, allowing for assessment of statistical significance. As mentioned earlier, in computer simulations, MPD showed no correlation with *S* and MNTD showed a negative correlation with *S.* In our data, the relationship between *S* and MPD was moderately positive, but there was only a weak negative correlation between *S* and MNTD. Again, this discrepancy may indicate a non-random change in phylogenetic diversity over the *S* gradient. Communities with high MPD and NRI values indicate species assemblages with ancient speciation events and possibly greater potential for evolutionary change that will allow populations to persist in changing environments. Communities with high MNTD and NTI values indicate species assemblages with more recent speciation events, which may indicate adaptive radiations that have resulted in endemic species, a site characteristic valued by conservation planners.

Abundance-weighted (AW) metrics can add value to biodiversity comparisons because they give an indication of the impact of evolutionary history on community assembly. When AW metric values are greater than the incidence metric values relative to a comparable community, this is an indication there are some species that may be dominant at a site. From the correlations reported in our results, the relationships between the species incidence metrics and the AW metrics confound diversity comparisons because the relative values at each site are not consistent such that sites with high abundance of some species may be identified. Our results may not lead to strong conclusions because most of the values are not statistically significant; however, this project represents the possibilities for calculating multiple PD metrics once a phylogeny is estimated. The value in calculating and comparing all of these metrics is to identify when empirical results do not match predictions. These situations will draw attention to notable discrepancies such as the PD metric variations between South and NMP (above), which have equivalent *S* values in our study or the correlations that do not match computer simulations. Additionally, comparing multiple metrics can provide supporting evidence about community assembly.

PD_SES_, NRI, and NTI (and their AW counterparts) reveal patterns of phylogenetic structure or community assembly (i.e. phylogenetic clustering or phylogenetic overdispersion/evenness) when values are statistically significant. Otherwise they indicate random assembly. All three metrics should result in the same characterization about species relatedness (Kembel et al. 2010). Only one of our results was statistically significant (NTI for NMP), indicating that the species at this site are phylogenetically clustered and assembled through environmental filtering (Cavender‐Bares et al. 2004, Pausas and Verdú 2010). Larger datasets should result in statistically significant values and show a clear pattern across sites leading to stronger conclusions about the phylogenetic structure of communities.

Calculating the phylogenetic signal of functional plant traits can also give an indication about a community through assembly of the traits in question. To test this component of biodiversity at our sites, we mapped specific leaf area (SLA) onto the phylogenetic tree and calculated the *K* statistic. Only one value was statistically different from Brownian motion – the *K* statistic for the South community was greater than one, indicating this trait is conserved across the tree and the species resemble each other more than expected by chance (low diversity). In the other communities, the values were not statistically significant and, therefore, indicate random trait assembly. Ideally for a study of trait evolution and indication of functional diversity at a site, more than one functional trait should be included and the relationship between the *K* statistic, *S*, and PD should be analyzed.

Since each metric characterizes biodiversity differently, it is important to choose the correct metric for the application as described above. No single metric considers all aspects of diversity and should be chosen based on the question of interest (Cadotte et al. 2010b). None of the traditional metrics consider evolutionary similarities or differences, but PD metrics can address fundamental species variation that contributes to healthy communities that have the ability to adapt to future environmental changes. For the most comprehensive characterization of biodiversity in a community, we recommend calculating all of these metrics. When large datasets are evaluated and resulting values are statistically significant, the various metrics should agree; when they do not, the metrics that vary should highlight the source of the discrepancies. When results are not statistically significant or when comparing single datasets between communities (as in our study), then non-parametric rank-based tests, such as a Kruskal-Wallis and Mann-Whitney can provide an indication of relative biodiversity. These rank-based tests allowed us to combine multiple metrics and get an overall sense of diversity at each site. The significant variation between NMP and NVP may be, in part, due to the great difference in geographical range sampled between the sites (2100 ha at NVP vs. 93 ha at NMP) but may also be due to variation in soil composition or historical land use (bison and cattle grazing at NVP vs. NMP, which has never been plowed or grazed). The comparatively low difference in diversity between the three sites within NVP may be due to fairly similar plant compositions and abiotic conditions.

### Conclusions

In one of the few empirical studies ever conducted that calculated the 17 most common PD metrics from massively parallel sequencing data, our results provide a baseline of data for future comparisons of biodiversity metrics. From this study, we drew five primary conclusions: 1) traditional indices do a fairly good job of quantifying overall diversity at a site, but to characterize the source of biodiversity such as ancient vs. recent speciation events, phylogenetic relationships must be incorporated; 2) *S* may be a good indicator for some PD metrics but not for others; 3) multiple diversity indices (both traditional and phylogenetic) should be calculated for a comprehensive biodiversity analysis; 4) inclusion of large species numbers (i.e. > 80 species) may be needed to obtain statistically significant results and to detect phylogenetic diversity beyond *S*; and 5) comparisons of PD metrics must be based on phylogenies estimated from equivalent character datasets. Future investigations are needed that 1) include larger numbers of taxa; 2) compare metrics between differing geographical sites; 3) include multiple traits for a comprehensive analysis of FD; and 4) compare PD metrics calculated from phylogenies estimated from various gene datasets (from three to many genes) to determine the effective number of genes necessary to calculate informative PD metrics. Our results, as well as future results, will contribute to the growing database of empirical PD metric data that will aid community ecologists and conservation biologists in future investigations of biodiversity and selection of priority regions for preservation.

## Supplementary Material

Supplementary material 1 Species list, Voucher numbers, and Specific Leaf Area Data Data type: PDF
Brief description: All species included in the study, herbarium voucher numbers, and average specific leaf area (SLA) calculated for each species
File: oo_46179.pdf S.K. Aust, D.L. Ahrendsen, and P.R. Kellar

Supplementary material 2 GenBank Accession Numbers Data type: PDF
Brief description: GenBank accession numbers for each gene/region by organelle.
Note: "-" indicates a missing gene
File: oo_46180.pdf S.K. Aust, D.L. Ahrendsen, and P.R. Kellar

Supplementary material 3 rbcL Phylogeny Data type: JPG
Brief description: Maximum likelihood (ML) tree (-ln L=10645.92) inferred from *rbcL* only (Suppl. material 7); matching 1 of 68 most parsimonious (MP) trees except were dagger (†) is shown. Tree includes 62 asterid species and 3 outgroups (*Comandra umbellata*, *Silene vulgaris*, and *Silene antirrhina*). Numbers above branches indicate branch lengths used to calculate various Phylogenetic Diversity (PD) metrics. Numbers below the branches indicate MP/ML bootstrap support values resulting from 1000 replicates each. Low branch support (<50) is indicated by an asterisk (*). Missing bootstrap values are denoted by a dash (-).
File: oo_44180.jpg S.K. Aust, D.L. Ahrendsen, and P.R. Kellar

Supplementary material 4 matK Phylogeny Data type: JPG
Brief description: Maximum likelihood (ML) tree (-ln L=19796.78) inferred from *matK* only (Suppl. material 8); matching 1 of 52 most parsimonious (MP) trees except were dagger (†) is shown. Tree includes 62 asterid species and 3 outgroups (*Comandra umbellata*, *Silene vulgaris*, and *Silene antirrhina*). Numbers above branches indicate branch lengths used to calculate various Phylogenetic Diversity (PD) metrics. Numbers below the branches indicate MP/ML bootstrap support values resulting from 1000 replicates each. Low branch support (<50) is indicated by an asterisk (*). Missing bootstrap values are denoted by a dash (-).
File: oo_44181.jpg S.K. Aust, D.L. Ahrendsen, P.R. Kellar

Supplementary material 5 rbcL + matK Phylogeny Data type: JPG
Brief description: Maximum likelihood (ML) tree (-ln L=30809.97) inferred from the concatenation of *rbcL* + *matK* (Suppl. material 9); matching one most parsimonious (MP) tree except were dagger (†) is shown. Tree includes 62 asterid species and 3 outgroups (*Comandra umbellata*, *Silene vulgaris*, and *Silene antirrhina*). Numbers above branches indicate branch lengths used to calculate various Phylogenetic Diversity (PD) metrics. Numbers below the branches indicate MP/ML bootstrap support values resulting from 1000 replicates each. Low branch support (<50) is indicated by an asterisk (*). Missing bootstrap values are denoted by a dash (-).
File: oo_44182.jpg S.K. Aust, D.L. Ahrendsen, P.R. Kellar

Supplementary material 6 cpmtnuc Nexus file Data type: Nexus
Brief description: Nexus alignment file.
File: oo_47041.nex S.K. Aust, D.L. Ahrendsen, P.R. Kellar

Supplementary material 7 rbcL Nexus file Data type: Nexus
Brief description: Nexus alignment file.
File: oo_47040.nex S.K. Aust, D.L. Ahrendsen, P.R. Kellar

Supplementary material 8 matK Nexus file Data type: Nexus
Brief description: Nexus alignment file.
File: oo_47042.nex S.K. Aust, D.L. Ahrendsen, P.R. Kellar

Supplementary material 9 rbcL + matK Nexus file Data type: Nexus
Brief description: Nexus alignment file.
File: oo_47043.nex S.K. Aust, D.L. Ahrendsen, P.R. Kellar

## Figures and Tables

**Table 1.** Summary of definitions, descriptions, software, and functions to calculate 17 phylogenetic diversity metrics, four traditional diversity indices, and the *K* statistic for the functional trait: specific leaf area.

Metric	Definition	Description	Software^a^	Citation
PD_Faith_	original PD metric	the sum of branch lengths between species in a tree	pd	Faith 1992
PD_SES_	standardized effect size of PD_Faith_	standardized effect size of PD vs. a null community	ses.pd	Webb et al. 2008
MPD^b^	mean pairwise distance	mean phylogenetic distance connecting species	mpd	Webb 2000
MNTD^b^	mean nearest taxon distance	mean phylogenetic distance for each species to its closest relative	mntd	Webb et al. 2002
NRI^b^	net relatedness index	MPD vs. a null community	ses.mpd	Webb 2000
NTI^b^	nearest taxon index	MNTD vs. a null community	ses.mntd	Webb 2000
SPD^​b^	sum of phylogenetic distances	sum of phylogenetic distances between pairs of species in a community	mpd* number of species pairs	Crozier 1997, Helmus et al. 2007, Vellend et al. 2011
PSV	phylogenetic species variability	related to NRI, but is independent of S	psv	Helmus et al. 2007
PSE	phylogenetic species evenness	variation of PSV but incorporates species abundance	pse	Helmus et al. 2007
PSC	phylogenetic species clustering	related to NTI, quantifies branch tip clustering of species in a tree	psc	Helmus et al. 2007
PSR	phylogenetic species richness	related to S and incorporates phylogenetic relatedness	psr	Helmus et al. 2007
I_ST_	local phylogenetic similarity excess	local phylogenetic similarity excess; average among-community diversity/total diversity across all samples	raoD	Hardy and Senterre 2007, Hardy and Jost 2008
*K*	measure of phylogenetic signal	a measure of the likeliness of phylogenetically related species to resemble each other	Kcalc	Blomberg et al. 2003
*S*	species richness	total number of species in a sampled site	-	Gotelli and Chao 2013
ENS	effective number of species	exponential of the Shannon-Weiner index; the number of species randomly generated for each community in order to equal the entropy for that community	EstimateS	Gotelli and Chao 2013
*S*_J_	Jaccard index; measure of similarity between sites	compares the number of shared species to the total number of species in the combined sites	EstimateS	Jaccard 1912, Jost 2006
*S*_S_	Sørensen index; measure of similarity between sites	applies weight to species common to each site over those found at only one site, and compares the number of shared species to the total number of species in the combined sites	EstimateS	Sorensen 1948, Jost 2006
^a^ - Metrics were calculated either in R (Version 3.0.1; R 2013) using the Picante package (Kembel et al. 2010) and the R function listed, or EstimateS (Version 9; Colwell 2013). ^b^ - Metrics with incidence and abundance-weighted versions				

**Table 2.** Alignment lengths and tree statistics for all datasets.

Tree/dataset	alignment length (bp)	Pairwise % identity	Tree/dataset length	# Parsimony informative characters	CI	RI
*matK*	1737	83.9%	3605	861	0.4638	0.7697
*rbcL*	1464	93.2%	1657	379	0.3744	0.7323
*rbcL* + *matK*	3192	87.9%	5265	1234	0.4325	0.7546
cpmtnuc^a^	65480	92.1%	70517	17823	0.4539	0.7718
cpmtnuc^a^: tree inferred from concatenation of 76 plastid genes, six mitochondrial genes, and three nuclear repeat regions Notes: Consistency index (CI) and retention index (RI) exclude uninformative characters. bp = nucleotide base-pairs; alignments were uploaded to the Dryad Digital Repository						

**Table 3.** Seventeen PD metrics calculated from the phylogeny inferred from 76 plastid genes, six mitochondrial genes, and three nuclear repeat regions (cpmtnuc), four traditional diversity indices, and the *K* statistic for one functional trait. Metrics were calculated for Nine-Mile Prairie (NMP), Niobrara Valley Preserve (NVP) and the three sites within NVP: North (N), South (So), and West (W).

Metric	NMP		South		West		North		NVP
PD_Faith_	0.535		0.625		0.914		0.964		1.280
PD_SES_	-1.317		-0.515		0.053		-0.554		0.621
MPD	0.097		0.089		0.102		0.097		0.104
MPD_aw_	0.077*		0.101		0.083		0.094		0.097*
MNTD	0.022*		0.035		0.029		0.025		0.025
MNTD_aw_	0.017*		0.055		0.021		0.030		0.030
NRI	0.592		1.264		-0.036		0.876		-0.610
NRI_aw_	0.863		-1.357		-0.285		-0.205		-0.534
NTI	2.039*		0.401		0.596		1.091		0.295
NTI_aw_	1.559		-0.799		0.565		-0.117		-0.382
SPD	22.322		20.468		57.376		75.523		154.517
SPD_aw_	17.776		23.267		46.443		73.007		143.874
PSV	0.441		0.358		0.416		0.396		0.422
PSE	0.356		0.383		0.329		0.372		0.375
PSC	0.888		0.858*		0.879		0.893		0.897
PSR	9.706		7.868		14.154		15.829		23.195
I_ST_	9M:NVP=0.009			N:S=0.008		N:W=0.005		S:W=0.007	
				9M:N=0.011		9M:W=0.013		9M:S=0.020	
K	0.154		1.171*		0.058		0.028		0.041
S	22		22		34		40		55
ENS	31.6		56.9		58.4		47.3		53.3
S_J_	9M:NVP=0.172			N:So=0.326		N:W=0.431		So:W=0.436	
				9M:N=0.200		9M:W=0.170		9M:So=0.075	
S_S_	9M:NVP=0.293			N:So=0.492		N:W=0.603		So:W=0.607	
				9M:N=0.333		9M:W=0.291		9M:So=0.140	
Notes: “*” indicates statistical significance (p< 0.05)									
