# Supplementary material for: Biodiversity assessment among two Nebraska prairies: a comparison between traditional and phylogenetic diversity indices
Source: Biodivers Data J. 2015 Jul 17;(3):e5403. doi: 10.3897/BDJ.3.e5403 (PMC4549632; doi:10.3897/BDJ.3.e5403)
Supplement: Supplementary material 1 — Species list, Voucher numbers, and Specific Leaf Area Data [file biodiversity_data_journal-3-e5403-s001.pdf]

Family	Species	Herbarium voucher #	SLA (cm^2/g)
Apiaceae	*Cicuta maculata*	Kellar1390	133.8
Apiaceae	*Conium maculatum*	Aust166	364.1
Apiaceae	*Zizia aurea*	Aust158	180.9
Apocynaceae	*Asclepias syriaca*	Aust167	150.2
Apocynaceae	*Asclepias verticillata*	Aust170	103.5
Asteraceae	*Achillea millefolium*	Steele1317*	120.2
Asteraceae	*Antennaria howellii* subsp. *neodioica*	Steele1262*	139.8
Asteraceae	*Antennaria negleta*	Steele1258*	162.4
Asteraceae	*Carduus nutans*	Aust168	161.4
Asteraceae	*Cirsium canescens*	Steele1302*	89.4
Asteraceae	*Cirsium undulatum*	Steele1355*	80.2
Asteraceae	*Echinacea angustifolia*	Steele1365*	79.5
Asteraceae	*Erigeron bellidiastrum*	Steele1299*	237.4
Asteraceae	*Erigeron philadelphicus*	Steele1323*	298.8
Asteraceae	*Erigeron strigosus*	Steele1357*	177.3
Asteraceae	*Grindelia squarrosa* var. *squarrosa*	Aust202	96.3
Asteraceae	*Gutierrezia sarothrae*	Steele1360*	96.0
Asteraceae	*Helianthus annuus*	Aust175	134.9
Asteraceae	*Helianthus pauciflorus* subsp. *subrhomboideus*	Aust197	58.8
Asteraceae	*Helianthus petiolaris*	Steele1366*	170.9
Asteraceae	*Helianthus tuberosus*	Aust189	107.5
Asteraceae	*Heliopsis helianthoides* var. *occidentalis*	Aust182	130.9
Asteraceae	*Heterotheca stenophylla* var. *stenophylla*	Steele1350*	136.7
Asteraceae	*Heterotheca villosa*	Kellar1379*	103.6
Asteraceae	*Hymenopappus tenuifolius*	Steele1325*	106.0
Asteraceae	*Lactuca ludoviciana*	Kellar1383*	320.8
Asteraceae	*Liatris glabrata*	Kellar1347*	71.4
Asteraceae	*Lygodesmia juncea*	Steele1362*	127.3
Asteraceae	*Ratibida columnifera*	Steele1354*	114.6
Asteraceae	*Rudbeckia hirta* var. *pulcherrima*	Aust172	160.4
Asteraceae	*Senecio integerrimus*	Steele1274*	324.5
Asteraceae	*Silphium integrifolum* var. *laeve*	Aust192	77.8
Asteraceae	*Solidago gigantea*	Kellar1389*	174.4
Asteraceae	*Solidago missouriensis*	Aust198	74.8
Asteraceae	*Thelesperma filifolium*	Steele1308*	142.3
Asteraceae	*Tragopogon dubius*	Steele1291*	255.9
Asteraceae	*Vernonia baldwinii*	Aust195	112.2
Asteraceae	*Xanthisma spinulosum*	Steele1352*	108.0
Balsaminaceae	*Impatiens capensis*	Kellar1393	773.9
Campanulaceae	*Campanula rotundifolia*	Steele1318	252.1
Caprifoliaceae	*Symphoricarpos occidentalis*	Steele1358	180.6
Caryophyllaceae	*Silene antirrhina*	Kellar1417	210.0
Caryophyllaceae	*Silene vulgaris*	Kellar1416	144.8
Convolvulaceae	*Convolvulus arvensis*	Kellar1382	261.8
Convolvulaceae	*Evolvulus nuttallianus*	Aust160	141.7
Convolvulaceae	*Ipomoea leptophylla*	Steele1349	174.7
Hydrophyllaceae	*Ellisia nyctelea*	Steele1275	258.9
Lamiaceae	*Monarda fistulosa* var. *mollis*	Aust190	231.8
Lamiaceae	*Nepeta cataria*	Aust177	295.4
Lamiaceae	*Salvia nemorosa*	Aust180	176.1
Lamiaceae	*Teucrium canadense*	Aust187	200.4
Plantaginaceae	*Plantago patagonica*	Steele1311	127.5
Primulaceae	*Androsace occidentalis*	Steele1279	238.1

Rubiaceae	*Galium aparine*	Steele1309	257.3
Santalaceae	*Comandra umbellata*	Kellar1411	166.3
Scrophulariaceae	*Penstemon angustifolius*	Steele1294	115.7
Scrophulariaceae	*Penstemon gracilis*	Aust173	113.5
Scrophulariaceae	*Verbascum thapsus*	Steele1373	163.7
Scrophulariaceae	*Veronica americana*	Steele1269	274.2
Solanaceae	*Physalis heterophylla*	Aust182	123.7
Solanaceae	*Physalis virginiana*	Aust157	235.9
Solanaceae	*Solanum carolinense*	Aust169	143.7
Solanaceae	*Solanum rostratum*	Aust178	167.1
Solanaceae	*Solanum triflorum*	Aust161	204.9
Verbenaceae	*Verbena hastata*	Steele1369	17.5

Notes: * indicate those taxa for which plastid genes were obtained from Kellar et al., 2015.
