# Supplementary material for: Biodiversity assessment among two Nebraska prairies: a comparison between traditional and phylogenetic diversity indices
Source: Biodivers Data J. 2015 Jul 17;(3):e5403. doi: 10.3897/BDJ.3.e5403 (PMC4549632; doi:10.3897/BDJ.3.e5403)
Supplement: Supplementary material 2 — GenBank Accession Numbers [file biodiversity_data_journal-3-e5403-s002.pdf]

GenBank accession numbers for each gene/region by organelle

Plastid (cpDNA) Page 1 of 5:

**Species**	atpA	atpB	atpE	atpF	atpH	atpI	ccsA	cemA	clpP	infA	matK	ndhA	ndhB	ndhC	ndhD
Androsace occidentalis	KT176193	KT176233	KT176273	KT176313	KT176353	KT176393	KT176433	KT176473	KT176513	--	KT176584	KT176624	KT176664	KT176704	KT176744
Asclepias verticillata	KT176194	KT176234	KT176274	KT176314	KT176354	KT176394	KT176434	KT176474	KT176547	KT176553	KT176585	KT176625	KT176665	KT176705	KT176745
Carduus nutans	KT176195	KT176235	KT176275	KT176315	KT176355	KT176395	KT176435	KT176475	KT176522	KT176554	KT176586	KT176627	KT176666	KT176706	KT176746
Vernonia baldwinii	KT176196	KT176241	KT176276	KT176316	KT176356	KT176396	KT176436	KT176476	KT176530	KT176562	KT176587	KT176628	KT176667	KT176707	KT176747
Helianthus pauciflorus	KT176197	KT176236	KT176277	KT176317	KT176357	KT176397	KT176437	KT176477	KT176525	KT176557	KT176588	KT176629	KT176668	KT176708	KT176748
Helianthus tuberosa	KT176198	KT176237	KT176278	KT176318	KT176358	KT176398	KT176438	KT176478	KT176526	KT176558	KT176589	KT176630	KT176669	KT176709	KT176749
Heliopsis helianthoides var occidentalis	KT176201	KT176240	KT176279	KT176319	KT176359	KT176399	KT176439	KT176479	KT176527	KT176561	KT176590	KT176631	KT176670	KT176710	KT176750
Rudbeckia hirta var pulcherrima	KT176200	KT176238	KT176280	KT176320	KT176360	KT176400	KT176440	KT176480	KT176529	KT176560	KT176591	KT176633	KT176671	KT176711	KT176751
Silphium integrifolum	KT176199	KT176239	KT176281	KT176321	KT176361	KT176401	KT176441	KT176481	KT176528	KT176559	KT176592	KT176632	KT176672	KT176712	KT176752
Grindelia squarrosa	KT176202	KT176242	KT176282	KT176322	KT176362	KT176402	KT176442	KT176482	KT176523	KT176555	KT176593	KT176634	KT176673	KT176713	KT176753
Solidago missouriensis	KT176203	KT176243	KT176283	KT176323	KT176363	KT176403	KT176443	KT176483	KT176524	KT176556	KT176594	KT176635	KT176674	KT176714	KT176754
Symphoricarpus occidentalis	KT176226	KT176247	KT176284	KT176324	KT176364	KT176404	KT176444	KT176484	KT176549	KT176583	KT176595	KT176639	KT176675	KT176715	KT176755
Cicuta maculata	KT176204	KT176244	KT176285	KT176325	KT176365	KT176405	KT176445	KT176485	KT176532	KT176571	KT176596	KT176636	KT176676	KT176716	KT176756
Conium maculatum	KT176205	KT176245	KT176286	KT176326	KT176366	KT176406	KT176446	KT176486	KT176533	KT176572	KT176597	KT176637	KT176677	KT176717	KT176757
Zizia aurea	KT176206	KT176246	KT176287	KT176327	KT176367	KT176407	KT176447	KT176487	KT176534	KT176573	KT176598	KT176638	KT176678	KT176718	KT176758
Ellisia nyctelea	KT176207	KT176248	KT176288	KT176328	KT176368	KT176408	KT176448	KT176488	KT176514	KT176574	KT176599	KT176640	KT176679	KT176719	KT176759
Physalis heterophylla	KT176208	KT176258	KT176289	KT176329	KT176369	KT176409	KT176449	KT176489	KT176539	KT176577	KT176600	KT176651	KT176680	KT176720	KT176760
Physalis virginiana	KT176209	KT176259	KT176290	KT176330	KT176370	KT176410	KT176450	KT176490	KT176540	KT176578	KT176601	KT176652	KT176681	KT176721	KT176761
Solanum carolinense	KT176210	KT176260	KT176291	KT176331	KT176371	KT176411	KT176451	KT176491	KT176542	KT176579	KT176602	KT176653	KT176682	KT176722	KT176762
Solanum rostratum	KT176211	KT176261	KT176292	KT176332	KT176372	KT176412	KT176452	KT176492	KT176543	KT176580	KT176603	KT176654	KT176683	KT176723	KT176763
Solanum triflorum	KT176212	KT176262	KT176293	KT176333	KT176373	KT176413	KT176453	KT176493	KT176541	KT176581	KT176604	KT176655	KT176684	KT176724	KT176764
Monarda fistulosa var mollis	KT176213	KT176249	KT176294	KT176334	KT176374	KT176414	KT176454	KT176494	KT176515	KT176563	KT176605	KT176641	KT176685	KT176725	KT176765
Nepeta cataria	KT176215	KT176250	KT176295	KT176335	KT176375	KT176415	KT176455	KT176495	KT176516	KT176564	KT176606	KT176642	KT176686	KT176726	KT176766
Salvia nemorosa	KT176214	KT176251	KT176296	KT176336	KT176376	KT176416	KT176456	KT176496	KT176517	KT176565	KT176607	KT176643	KT176687	KT176727	KT176767
Teucrium canadense	KT176218	KT176255	KT176297	KT176337	KT176377	KT176417	KT176457	KT176497	KT176537	KT176568	KT176608	KT176647	KT176688	KT176728	KT176768
Verbascum thapsus	KT176219	KT176254	KT176298	KT176338	KT176378	KT176418	KT176458	KT176498	KT176519	KT176569	KT176609	KT176646	KT176689	KT176729	KT176769
Penstemon angustifolius	KT176216	KT176252	KT176299	KT176339	KT176379	KT176419	KT176459	KT176499	KT176520	KT176566	KT176610	KT176644	KT176690	KT176730	KT176770
Penstemon gracilis	KT176217	KT176253	KT176300	KT176340	KT176380	KT176420	KT176460	KT176500	KT176521	KT176567	KT176611	KT176645	KT176691	KT176731	KT176771
Verbena hastata	KT176220	KT176256	KT176301	KT176341	KT176381	KT176421	KT176461	KT176501	KT176551	KT176582	KT176612	KT176648	KT176692	KT176732	KT176772
Veronica americana	KT176221	KT176257	KT176302	KT176342	KT176382	KT176422	KT176462	KT176502	KT176518	KT176570	KT176613	KT176649	KT176693	KT176733	KT176773
Plantago patagonica	KT176222	KT176267	KT176303	KT176343	KT176383	KT176423	KT176463	KT176503	KT176550	KT176575	KT176614	KT176650	KT176694	KT176734	KT176774
Impatiens capensis	KT176228	KT176266	KT176304	KT176344	KT176384	KT176424	KT176464	KT176504	KT176535	KT176576	KT176615	KT176656	KT176695	KT176735	KT176775
Convolvulus arvensis	KT176223	KT176263	KT176305	KT176345	KT176385	KT176425	KT176465	KT176505	KT176544	--	KT176616	KT176660	KT176696	KT176736	KT176776
Ipomoea leptophylla	KT176224	KT176264	KT176306	KT176346	KT176386	KT176426	KT176466	KT176506	KT176545	--	KT176617	KT176661	KT176697	KT176737	KT176777
Evolvulus nuttallianus	KT176225	KT176265	KT176307	KT176347	KT176387	KT176427	KT176467	KT176507	KT176548	--	KT176618	KT176662	KT176698	KT176738	KT176778
Comandra umbellata	KT176227	KT176268	KT176308	KT176348	KT176388	KT176428	KT176468	KT176508	KT176538	--	KT176619	KT176657	KT176699	KT176739	KT176779
Galium aparine	KT176231	KT176272	KT176309	KT176349	KT176389	KT176429	KT176469	KT176509	KT176531	--	KT176620	KT176663	KT176700	KT176740	KT176780
Silene antirrhina	KT176229	KT176269	KT176310	KT176350	KT176390	KT176430	KT176470	KT176510	KT176546	--	KT176621	KT176658	KT176701	KT176741	KT176781
Silene vulgaris	KT176230	KT176270	KT176311	KT176351	KT176391	KT176431	KT176471	KT176511	KT176536	--	KT176622	KT176659	KT176702	KT176742	KT176782
Campanula rotundifolia	KT176232	KT176271	KT176312	KT176352	KT176392	KT176432	KT176472	KT176512	KT176552	--	KT176623	KT176626	KT176703	KT176743	KT176783

Plastid (cpDNA) Page 2 of 5:

**Species**	ndhE	ndhF	ndhG	ndhH	ndhI	ndhJ	ndhK	petA	petB	petD	petG	petL	petN	psaA	psaB
Androsace occidentalis	KT176784	KT176824	KT176864	KT176904	KT176944	KT176984	KT177024	KT177064	KT177104	KT177144	KT177184	KT177224	KT177264	KT177304	KT177344
Asclepias verticillata	KT176785	KT176825	KT176865	KT176905	KT176945	KT176985	KT177025	KT177065	KT177105	KT177145	KT177185	KT177225	KT177265	KT177305	KT177345
Carduus nutans	KT176786	KT176826	KT176866	KT176906	KT176946	KT176986	KT177026	KT177066	KT177106	KT177146	KT177186	KT177226	KT177266	KT177306	KT177346
Vernonia baldwinii	KT176787	KT176827	KT176867	KT176907	KT176947	KT176987	KT177027	KT177067	KT177107	KT177147	KT177187	KT177227	KT177267	KT177307	KT177347
Helianthus pauciflorus	KT176788	KT176828	KT176868	KT176908	KT176948	KT176988	KT177028	KT177068	KT177108	KT177148	KT177188	KT177228	KT177268	KT177308	KT177348
Helianthus tuberosa	KT176789	KT176829	KT176869	KT176909	KT176949	KT176989	KT177029	KT177069	KT177109	KT177149	KT177189	KT177229	KT177269	KT177309	KT177349
Heliopsis helianthoides var occidentalis	KT176790	KT176830	KT176870	KT176910	KT176950	KT176990	KT177030	KT177072	KT177112	KT177152	KT177192	KT177232	KT177272	KT177312	KT177352
Rudbeckia hirta var pulcherrima	KT176791	KT176831	KT176871	KT176911	KT176951	KT176991	KT177031	KT177070	KT177110	KT177150	KT177190	KT177230	KT177270	KT177310	KT177350
Silphium integrifolum	KT176792	KT176832	KT176872	KT176912	KT176952	KT176992	KT177032	KT177071	KT177111	KT177151	KT177191	KT177231	KT177271	KT177311	KT177351
Grindelia squarrosa	KT176793	KT176833	KT176873	KT176913	KT176953	KT176993	KT177033	KT177073	KT177113	KT177153	KT177193	KT177233	KT177273	KT177313	KT177353
Solidago missouriensis	KT176794	KT176834	KT176874	KT176914	KT176954	KT176994	KT177034	KT177074	KT177114	KT177154	KT177194	KT177234	KT177274	KT177314	KT177354
Symphoricarpus occidentalis	KT176795	KT176835	KT176875	KT176915	KT176955	KT176995	KT177035	KT177078	KT177118	KT177158	KT177198	KT177238	KT177278	KT177318	KT177358
Cicuta maculata	KT176796	KT176836	KT176876	KT176916	KT176956	KT176996	KT177036	KT177075	KT177115	KT177155	KT177195	KT177235	KT177275	KT177315	KT177355
Conium maculatum	KT176797	KT176837	KT176877	KT176917	KT176957	KT176997	KT177037	KT177076	KT177116	KT177156	KT177196	KT177236	KT177276	KT177316	KT177356
Zizia aurea	KT176798	KT176838	KT176878	KT176918	KT176958	KT176998	KT177038	KT177077	KT177117	KT177157	KT177197	KT177237	KT177277	KT177317	KT177357
Ellisia nyctelea	KT176799	KT176839	KT176879	KT176919	KT176959	KT176999	KT177039	KT177079	KT177119	KT177159	KT177199	KT177239	KT177279	KT177319	KT177359
Physalis heterophylla	KT176800	KT176840	KT176880	KT176920	KT176960	KT177000	KT177040	KT177080	KT177120	KT177160	KT177200	KT177240	KT177280	KT177320	KT177360
Physalis virginiana	KT176801	KT176841	KT176881	KT176921	KT176961	KT177001	KT177041	KT177081	KT177121	KT177161	KT177201	KT177241	KT177281	KT177321	KT177361
Solanum carolinense	KT176802	KT176842	KT176882	KT176922	KT176962	KT177002	KT177042	KT177082	KT177122	KT177162	KT177202	KT177242	KT177282	KT177322	KT177362
Solanum rostratum	KT176803	KT176843	KT176883	KT176923	KT176963	KT177003	KT177043	KT177083	KT177123	KT177163	KT177203	KT177243	KT177283	KT177323	KT177363
Solanum triflorum	KT176804	KT176844	KT176884	KT176924	KT176964	KT177004	KT177044	KT177084	KT177124	KT177164	KT177204	KT177244	KT177284	KT177324	KT177364
Monarda fistulosa var mollis	KT176805	KT176845	KT176885	KT176925	KT176965	KT177005	KT177045	KT177085	KT177125	KT177165	KT177205	KT177245	KT177285	KT177325	KT177365
Nepeta cataria	KT176806	KT176846	KT176886	KT176926	KT176966	KT177006	KT177046	KT177087	KT177127	KT177167	KT177207	KT177247	KT177287	KT177327	KT177367
Salvia nemorosa	KT176807	KT176847	KT176887	KT176927	KT176967	KT177007	KT177047	KT177086	KT177126	KT177166	KT177206	KT177246	KT177286	KT177326	KT177366
Teucrium canadense	KT176808	KT176848	KT176888	KT176928	KT176968	KT177008	KT177048	KT177091	KT177131	KT177171	KT177211	KT177251	KT177291	KT177331	KT177371
Verbascum thapsus	KT176809	KT176849	KT176889	KT176929	KT176969	KT177009	KT177049	KT177090	KT177130	KT177170	KT177210	KT177250	KT177290	KT177330	KT177370
Penstemon angustifolius	KT176810	KT176850	KT176890	KT176930	KT176970	KT177010	KT177050	KT177088	KT177128	KT177168	KT177208	KT177248	KT177288	KT177328	KT177368
Penstemon gracilis	KT176811	KT176851	KT176891	KT176931	KT176971	KT177011	KT177051	KT177089	KT177129	KT177169	KT177209	KT177249	KT177289	KT177329	KT177369
Verbena hastata	KT176812	KT176852	KT176892	KT176932	KT176972	KT177012	KT177052	KT177092	KT177132	KT177172	KT177212	KT177252	KT177292	KT177332	KT177372
Veronica americana	KT176813	KT176853	KT176893	KT176933	KT176973	KT177013	KT177053	KT177093	KT177133	KT177173	KT177213	KT177253	KT177293	KT177333	KT177373
Plantago patagonica	KT176814	KT176854	KT176894	KT176934	KT176974	KT177014	KT177054	KT177094	KT177134	KT177174	KT177214	KT177254	KT177294	KT177334	KT177374
Impatiens capensis	KT176815	KT176855	KT176895	KT176935	KT176975	KT177015	KT177055	KT177098	KT177138	KT177178	KT177218	KT177258	KT177298	KT177338	KT177378
Convolvulus arvensis	KT176816	KT176856	KT176896	KT176936	KT176976	KT177016	KT177056	KT177095	KT177135	KT177175	KT177215	KT177255	KT177295	KT177335	KT177375
Ipomoea leptophylla	KT176817	KT176857	KT176897	KT176937	KT176977	KT177017	KT177057	KT177096	KT177136	KT177176	KT177216	KT177256	KT177296	KT177336	KT177376
Evolvulus nuttallianus	KT176818	KT176858	KT176898	KT176938	KT176978	KT177018	KT177058	KT177097	KT177137	KT177177	KT177217	KT177257	KT177297	KT177337	KT177377
Comandra umbellata	KT176819	KT176859	KT176899	KT176939	KT176979	KT177019	KT177059	KT177099	KT177139	KT177179	KT177219	KT177259	KT177299	KT177339	KT177379
Galium aparine	KT176820	KT176860	KT176900	KT176940	KT176980	KT177020	KT177060	KT177100	KT177140	KT177180	KT177220	KT177260	KT177300	KT177340	KT177380
Silene antirrhina	KT176821	KT176861	KT176901	KT176941	KT176981	KT177021	KT177061	KT177101	KT177141	KT177181	KT177221	KT177261	KT177301	KT177341	KT177381
Silene vulgaris	KT176822	KT176862	KT176902	KT176942	KT176982	KT177022	KT177062	KT177102	KT177142	KT177182	KT177222	KT177262	KT177302	KT177342	KT177382
Campanula rotundifolia	KT176823	KT176863	KT176903	KT176943	KT176983	KT177023	KT177063	KT177103	KT177143	KT177183	KT177223	KT177263	KT177303	KT177343	KT177383

Plastid (cpDNA) Page 3 of 5:

Species	psaC	psaI	psaJ	psbA	psbB	psbC	psbD	psbE	psbF	psbH	psbI	psbJ	psbK	psbL	psbM
Androsace occidentalis	KT177384	KT177424	KT177464	KT177504	KT177544	KT177584	KT177624	KT177664	KT177704	KT177744	KT177784	KT177824	KT177864	KT177904	KT177944
Asclepias verticillata	KT177385	KT177425	KT177465	KT177505	KT177545	KT177585	KT177625	KT177665	KT177705	KT177745	KT177785	KT177825	KT177865	KT177905	KT177945
Carduus nutans	KT177386	KT177426	KT177466	KT177506	KT177546	KT177586	KT177626	KT177666	KT177706	KT177746	KT177786	KT177826	KT177866	KT177906	KT177946
Vernonia baldwinii	KT177387	KT177427	KT177467	KT177507	KT177547	KT177587	KT177627	KT177667	KT177707	KT177747	KT177787	KT177827	KT177867	KT177907	KT177947
Helianthus pauciflorus	KT177388	KT177428	KT177468	KT177508	KT177548	KT177588	KT177628	KT177668	KT177708	KT177748	KT177788	KT177828	KT177868	KT177908	KT177948
Helianthus tuberosa	KT177389	KT177429	KT177469	KT177509	KT177549	KT177589	KT177629	KT177669	KT177709	KT177749	KT177789	KT177829	KT177869	KT177909	KT177949
Heliopsis helianthoides var occidentalis	KT177392	KT177432	KT177472	KT177512	KT177552	KT177592	KT177632	KT177672	KT177712	KT177752	KT177792	KT177832	KT177872	KT177912	KT177952
Rudbeckia hirta var pulcherrima	KT177390	KT177430	KT177470	KT177510	KT177550	KT177590	KT177630	KT177670	KT177710	KT177750	KT177790	KT177830	KT177870	KT177910	KT177950
Silphium integrifolum	KT177391	KT177431	KT177471	KT177511	KT177551	KT177591	KT177631	KT177671	KT177711	KT177751	KT177791	KT177831	KT177871	KT177911	KT177951
Grindelia squarrosa	KT177393	KT177433	KT177473	KT177513	KT177553	KT177593	KT177633	KT177673	KT177713	KT177753	KT177793	KT177833	KT177873	KT177913	KT177953
Solidago missouriensis	KT177394	KT177434	KT177474	KT177514	KT177554	KT177594	KT177634	KT177674	KT177714	KT177754	KT177794	KT177834	KT177874	KT177914	KT177954
Symphoricarpus occidentalis	KT177398	KT177438	KT177478	KT177518	KT177558	KT177598	KT177638	KT177678	KT177718	KT177758	KT177798	KT177838	KT177878	KT177918	KT177958
Cicuta maculata	KT177395	KT177435	KT177475	KT177515	KT177555	KT177595	KT177635	KT177675	KT177715	KT177755	KT177795	KT177835	KT177875	KT177915	KT177955
Conium maculatum	KT177396	KT177436	KT177476	KT177516	KT177556	KT177596	KT177636	KT177676	KT177716	KT177756	KT177796	KT177836	KT177876	KT177916	KT177956
Zizia aurea	KT177397	KT177437	KT177477	KT177517	KT177557	KT177597	KT177637	KT177677	KT177717	KT177757	KT177797	KT177837	KT177877	KT177917	KT177957
Ellisia nyctelea	KT177399	KT177439	KT177479	KT177519	KT177559	KT177599	KT177639	KT177679	KT177719	KT177759	KT177799	KT177839	KT177879	KT177919	KT177959
Physalis heterophylla	KT177400	KT177440	KT177480	KT177520	KT177560	KT177600	KT177640	KT177680	KT177720	KT177760	KT177800	KT177840	KT177880	KT177920	KT177960
Physalis virginiana	KT177401	KT177441	KT177481	KT177521	KT177561	KT177601	KT177641	KT177681	KT177721	KT177761	KT177801	KT177841	KT177881	KT177921	KT177961
Solanum carolinense	KT177402	KT177442	KT177482	KT177522	KT177562	KT177602	KT177642	KT177682	KT177722	KT177762	KT177802	KT177842	KT177882	KT177922	KT177962
Solanum rostratum	KT177403	KT177443	KT177483	KT177523	KT177563	KT177603	KT177643	KT177683	KT177723	KT177763	KT177803	KT177843	KT177883	KT177923	KT177963
Solanum triflorum	KT177404	KT177444	KT177484	KT177524	KT177564	KT177604	KT177644	KT177684	KT177724	KT177764	KT177804	KT177844	KT177884	KT177924	KT177964
Monarda fistulosa var mollis	KT177405	KT177445	KT177485	KT177525	KT177565	KT177605	KT177645	KT177685	KT177725	KT177765	KT177805	KT177845	KT177885	KT177925	KT177965
Nepeta cataria	KT177407	KT177447	KT177487	KT177527	KT177567	KT177607	KT177647	KT177687	KT177727	KT177767	KT177807	KT177847	KT177887	KT177927	KT177967
Salvia nemorosa	KT177406	KT177446	KT177486	KT177526	KT177566	KT177606	KT177646	KT177686	KT177726	KT177766	KT177806	KT177846	KT177886	KT177926	KT177966
Teucrium canadense	KT177411	KT177451	KT177491	KT177531	KT177571	KT177611	KT177651	KT177691	KT177731	KT177771	KT177811	KT177851	KT177891	KT177931	KT177971
Verbascum thapsus	KT177410	KT177450	KT177490	KT177530	KT177570	KT177610	KT177650	KT177690	KT177730	KT177770	KT177810	KT177850	KT177890	KT177930	KT177970
Penstemon angustifolius	KT177408	KT177448	KT177488	KT177528	KT177568	KT177608	KT177648	KT177688	KT177728	KT177768	KT177808	KT177848	KT177888	KT177928	KT177968
Penstemon gracilis	KT177409	KT177449	KT177489	KT177529	KT177569	KT177609	KT177649	KT177689	KT177729	KT177769	KT177809	KT177849	KT177889	KT177929	KT177969
Verbena hastata	KT177412	KT177452	KT177492	KT177532	KT177572	KT177612	KT177652	KT177692	KT177732	KT177772	KT177812	KT177852	KT177892	KT177932	KT177972
Veronica americana	KT177413	KT177453	KT177493	KT177533	KT177573	KT177613	KT177653	KT177693	KT177733	KT177773	KT177813	KT177853	KT177893	KT177933	KT177973
Plantago patagonica	KT177414	KT177454	KT177494	KT177534	KT177574	KT177614	KT177654	KT177694	KT177734	KT177774	KT177814	KT177854	KT177894	KT177934	KT177974
Impatiens capensis	KT177418	KT177458	KT177498	KT177538	KT177578	KT177618	KT177658	KT177698	KT177738	KT177778	KT177818	KT177858	KT177898	KT177938	KT177978
Convolvulus arvensis	KT177415	KT177455	KT177495	KT177535	KT177575	KT177615	KT177655	KT177695	KT177735	KT177775	KT177815	KT177855	KT177895	KT177935	KT177975
Ipomoea leptophylla	KT177416	KT177456	KT177496	KT177536	KT177576	KT177616	KT177656	KT177696	KT177736	KT177776	KT177816	KT177856	KT177896	KT177936	KT177976
Evolvulus nuttallianus	KT177417	KT177457	KT177497	KT177537	KT177577	KT177617	KT177657	KT177697	KT177737	KT177777	KT177817	KT177857	KT177897	KT177937	KT177977
Comandra umbellata	KT177419	KT177459	KT177499	KT177539	KT177579	KT177619	KT177659	KT177699	KT177739	KT177779	KT177819	KT177859	KT177899	KT177939	KT177979
Galium aparine	KT177420	KT177460	KT177500	KT177540	KT177580	KT177620	KT177660	KT177700	KT177740	KT177780	KT177820	KT177860	KT177900	KT177940	KT177980
Silene antirrhina	KT177421	KT177461	KT177501	KT177541	KT177581	KT177621	KT177661	KT177701	KT177741	KT177781	KT177821	KT177861	KT177901	KT177941	KT177981
Silene vulgaris	KT177422	KT177462	KT177502	KT177542	KT177582	KT177622	KT177662	KT177702	KT177742	KT177782	KT177822	KT177862	KT177902	KT177942	KT177982
Campanula rotundifolia	KT177423	KT177463	KT177503	KT177543	KT177583	KT177623	KT177663	KT177703	KT177743	KT177783	KT177823	KT177863	KT177903	KT177943	KT177983

Species	psbN	psbT	psbZ	rbcL	rpl2	rpl14	rpl16	rpl20	rpl22	rpl23	rpl32	rpl33	rpl36	rpoA	rpoB
Androsace occidentalis	KT177984	KT178024	KT178064	KT178104	KT178144	KT178184	KT178224	KT178264	KT178304	KT178343	KT178379	KT178419	KT178458	KT178498	KT178538
Asclepias verticillata	KT177985	KT178025	KT178065	KT178105	KT178145	KT178185	KT178225	KT178296	KT178305	KT178344	KT178380	KT178420	KT178459	KT178520	KT178539
Carduus nutans	KT177986	KT178026	KT178066	KT178106	KT178146	KT178186	KT178226	KT178265	KT178324	KT178345	KT178381	KT178421	KT178460	KT178523	KT178565
Vernonia baldwinii	KT177987	KT178027	KT178067	KT178107	KT178147	KT178187	KT178227	KT178273	KT178325	KT178346	KT178382	KT178422	KT178461	KT178530	KT178573
Helianthus pauciflorus	KT177988	KT178028	KT178068	KT178108	KT178148	KT178188	KT178228	KT178268	KT178326	KT178347	KT178383	KT178423	KT178462	KT178526	KT178568
Helianthus tuberosa	KT177989	KT178029	KT178069	KT178109	KT178149	KT178189	KT178229	KT178269	KT178327	KT178348	KT178384	KT178424	KT178463	KT178527	KT178569
Heliopsis helianthoides var occidentalis	KT177992	KT178032	KT178072	KT178112	KT178152	KT178192	KT178232	KT178270	KT178330	KT178351	KT178385	KT178427	KT178466	KT178531	KT178572
Rudbeckia hirta var pulcherrima	KT177990	KT178030	KT178070	KT178110	KT178150	KT178190	KT178230	KT178271	KT178328	KT178349	KT178386	KT178425	KT178464	KT178529	KT178571
Silphium integrifolum	KT177991	KT178031	KT178071	KT178111	KT178151	KT178191	KT178231	KT178272	KT178329	KT178350	KT178387	KT178426	KT178465	KT178528	KT178570
Grindelia squarrosa	KT177993	KT178033	KT178073	KT178113	KT178153	KT178193	KT178233	KT178266	KT178331	KT178352	KT178388	KT178428	KT178467	KT178524	KT178566
Solidago missouriensis	KT177994	KT178034	KT178074	KT178114	KT178154	KT178194	KT178234	KT178267	KT178332	KT178353	KT178389	KT178429	KT178468	KT178525	KT178567
Symphoricarpus occidentalis	KT177998	KT178038	KT178078	KT178118	KT178158	KT178198	KT178238	KT178301	KT178333	KT178357	KT178393	KT178433	KT178472	KT178502	KT178562
Cicuta maculata	KT177995	KT178035	KT178075	KT178115	KT178155	KT178195	KT178235	KT178278	KT178334	KT178354	KT178390	KT178430	KT178469	KT178499	KT178540
Conium maculatum	KT177996	KT178036	KT178076	KT178116	KT178156	KT178196	KT178236	KT178279	KT178335	KT178355	KT178391	KT178431	KT178470	KT178500	KT178541
Zizia aurea	KT177997	KT178037	KT178077	KT178117	KT178157	KT178197	KT178237	KT178280	KT178336	KT178356	KT178392	KT178432	KT178471	KT178501	KT178542
Ellisia nyctelea	KT177999	KT178039	KT178079	KT178119	KT178159	KT178199	KT178239	KT178274	KT178306	KT178358	KT178394	KT178434	KT178473	KT178506	KT178546
Physalis heterophylla	KT178000	KT178040	KT178080	KT178120	KT178160	KT178200	KT178240	KT178289	KT178307	KT178359	KT178407	KT178435	KT178474	KT178507	KT178547
Physalis virginiana	KT178001	KT178041	KT178081	KT178121	KT178161	KT178201	KT178241	KT178290	KT178308	KT178360	KT178408	KT178436	KT178475	KT178508	KT178548
Solanum carolinense	KT178002	KT178042	KT178082	KT178122	KT178162	KT178202	KT178242	KT178275	KT178309	KT178361	KT178409	KT178437	KT178476	KT178509	KT178549
Solanum rostratum	KT178003	KT178043	KT178083	KT178123	KT178163	KT178203	KT178243	KT178276	KT178310	KT178362	KT178410	KT178438	KT178477	KT178510	KT178550
Solanum triflorum	KT178004	KT178044	KT178084	KT178124	KT178164	KT178204	KT178244	KT178277	KT178311	KT178363	KT178411	KT178439	KT178478	KT178511	KT178551
Monarda fistulosa var mollis	KT178005	KT178045	KT178085	KT178125	KT178165	KT178205	KT178245	KT178281	KT178314	KT178364	KT178396	KT178440	KT178479	KT178512	KT178552
Nepeta cataria	KT178007	KT178047	KT178087	KT178127	KT178167	KT178207	KT178247	KT178282	KT178316	KT178366	KT178398	KT178442	KT178481	KT178513	KT178554
Salvia nemorosa	KT178006	KT178046	KT178086	KT178126	KT178166	KT178206	KT178246	KT178283	KT178315	KT178365	KT178397	KT178441	KT178480	KT178514	KT178553
Teucrium canadense	KT178011	KT178051	KT178091	KT178131	KT178171	KT178211	KT178251	KT178288	KT178317	KT178370	KT178401	KT178446	KT178485	KT178519	KT178558
Verbascum thapsus	KT178010	KT178050	KT178090	KT178130	KT178170	KT178210	KT178250	KT178286	KT178318	KT178369	KT178402	KT178445	KT178484	KT178517	KT178557
Penstemon angustifolius	KT178008	KT178048	KT178088	KT178128	KT178168	KT178208	KT178248	KT178284	KT178312	KT178367	KT178399	KT178443	KT178482	KT178515	KT178555
Penstemon gracilis	KT178009	KT178049	KT178089	KT178129	KT178169	KT178209	KT178249	KT178285	KT178313	KT178368	KT178400	KT178444	KT178483	KT178516	KT178556
Verbena hastata	KT178012	KT178052	KT178092	KT178132	KT178172	KT178212	KT178252	KT178294	KT178319	KT178371	KT178404	KT178447	KT178486	KT178518	KT178559
Veronica americana	KT178013	KT178053	KT178093	KT178133	KT178173	KT178213	KT178253	KT178287	KT178323	KT178372	KT178405	KT178448	KT178487	KT178521	KT178560
Plantago patagonica	KT178014	KT178054	KT178094	KT178134	KT178174	KT178214	KT178254	KT178302	KT178339	KT178373	KT178403	KT178449	KT178488	KT178533	KT178561
Impatiens capensis	KT178018	KT178058	KT178098	KT178138	KT178178	KT178218	KT178258	KT178295	KT178338	KT178377	KT178395	KT178453	KT178492	KT178532	KT178563
Convolvulus arvensis	KT178015	KT178055	KT178095	KT178135	KT178175	KT178215	KT178255	KT178291	KT178320	KT178374	KT178414	KT178450	KT178489	KT178503	KT178543
Ipomoea leptophylla	KT178016	KT178056	KT178096	KT178136	KT178176	KT178216	KT178256	KT178292	KT178321	KT178375	KT178415	KT178451	KT178490	KT178504	KT178544
Evolvulus nuttallianus	KT178017	KT178057	KT178097	KT178137	KT178177	KT178217	KT178257	KT178293	KT178322	KT178376	KT178417	KT178452	KT178491	KT178505	KT178545
Comandra umbellata	KT178019	KT178059	KT178099	KT178139	KT178179	KT178219	KT178259	KT178300	--	--	KT178416	KT178454	KT178493	KT178522	KT178564
Galium aparine	KT178020	KT178060	KT178100	KT178140	KT178180	KT178220	KT178260	KT178297	KT178337	KT178378	KT178406	--	KT178494	KT178534	KT178577
Silene antirrhina	KT178021	KT178061	KT178101	KT178141	KT178181	KT178221	KT178261	KT178298	KT178341	--	KT178412	KT178455	KT178495	KT178536	KT178575
Silene vulgaris	KT178022	KT178062	KT178102	KT178142	KT178182	KT178222	KT178262	KT178299	KT178342	--	KT178413	KT178456	KT178496	KT178537	KT178576
Campanula rotundifolia	KT178023	KT178063	KT178103	KT178143	KT178183	KT178223	KT178263	KT178303	KT178340	--	KT178418	KT178457	KT178497	KT178535	KT178574

Plastid (cpDNA) Page 5 of 5:

Species	rpoC1	rpoC2	rps2	rps3	rps4	rps7	rps8	rps11	rps12	rps14	rps15	rps16	rps18	rps19	ycf3	ycf4
Androsace occidentalis	KT178578	KT178618	KT178658	KT178698	KT178738	KT178778	KT178818	KT178858	KT178898	KT178938	KT178978	KT179018	KT179057	KT179097	KT179137	KT179177
Asclepias verticillata	KT178579	KT178619	KT178691	KT178699	KT178739	KT178779	KT178819	KT178859	KT178899	KT178939	KT178979	KT179019	KT179095	KT179098	KT179138	KT179178
Carduus nutans	KT178608	KT178644	KT178659	KT178700	KT178740	KT178780	KT178820	KT178860	KT178900	KT178940	KT178980	KT179020	KT179058	KT179099	KT179139	KT179179
Vernonia baldwinii	KT178616	KT178652	KT178667	KT178701	KT178741	KT178781	KT178821	KT178861	KT178901	KT178941	KT178981	KT179021	KT179059	KT179100	KT179140	KT179180
Helianthus pauciflorus	KT178611	KT178647	KT178660	KT178702	KT178742	KT178782	KT178822	KT178862	KT178902	KT178942	KT178982	KT179022	KT179060	KT179101	KT179141	KT179181
Helianthus tuberosa	KT178612	KT178648	KT178661	KT178703	KT178743	KT178783	KT178823	KT178863	KT178903	KT178943	KT178983	KT179023	KT179061	KT179102	KT179142	KT179182
Heliopsis helianthoides var occidentalis	KT178615	KT178651	KT178664	KT178706	KT178746	KT178786	KT178826	KT178866	KT178906	KT178946	KT178986	KT179026	KT179062	KT179105	KT179145	KT179185
Rudbeckia hirta var pulcherrima	KT178613	KT178649	KT178662	KT178704	KT178744	KT178784	KT178824	KT178864	KT178904	KT178944	KT178984	KT179024	KT179064	KT179103	KT179143	KT179183
Silphium integrifolum	KT178614	KT178650	KT178663	KT178705	KT178745	KT178785	KT178825	KT178865	KT178905	KT178945	KT178985	KT179025	KT179063	KT179104	KT179144	KT179184
Grindelia squarrosa	KT178609	KT178645	KT178665	KT178707	KT178747	KT178787	KT178827	KT178867	KT178907	KT178947	KT178987	KT179027	KT179065	KT179106	KT179146	KT179186
Solidago missouriensis	KT178610	KT178646	KT178666	KT178708	KT178748	KT178788	KT178828	KT178868	KT178908	KT178948	KT178988	KT179028	KT179066	KT179107	KT179147	KT179187
Symphoricarpus occidentalis	KT178602	KT178623	KT178668	KT178712	KT178752	KT178792	KT178832	KT178872	KT178912	KT178952	KT178992	KT179032	KT179091	KT179111	KT179151	KT179191
Cicuta maculata	KT178599	KT178620	KT178683	KT178709	KT178749	KT178789	KT178829	KT178869	KT178909	KT178949	KT178989	KT179029	KT179082	KT179108	KT179148	KT179188
Conium maculatum	KT178600	KT178621	KT178684	KT178710	KT178750	KT178790	KT178830	KT178870	KT178910	KT178950	KT178990	KT179030	KT179083	KT179109	KT179149	KT179189
Zizia aurea	KT178601	KT178622	KT178685	KT178711	KT178751	KT178791	KT178831	KT178871	KT178911	KT178951	KT178991	KT179031	KT179084	KT179110	KT179150	KT179190
Ellisia nyctelea	KT178583	KT178624	KT178669	KT178713	KT178753	KT178793	KT178833	KT178873	KT178913	KT178953	KT178993	KT179033	KT179067	KT179112	KT179152	KT179192
Physalis heterophylla	KT178593	KT178625	KT178670	KT178714	KT178754	KT178794	KT178834	KT178874	KT178914	KT178954	KT178994	KT179034	KT179075	KT179113	KT179153	KT179193
Physalis virginiana	KT178594	KT178626	KT178671	KT178715	KT178755	KT178795	KT178835	KT178875	KT178915	KT178955	KT178995	KT179035	KT179076	KT179114	KT179154	KT179194
Solanum carolinense	KT178596	KT178627	KT178672	KT178716	KT178756	KT178796	KT178836	KT178876	KT178916	KT178956	KT178996	KT179036	KT179078	KT179115	KT179155	KT179195
Solanum rostratum	KT178597	KT178628	KT178673	KT178717	KT178757	KT178797	KT178837	KT178877	KT178917	KT178957	KT178997	KT179037	KT179079	KT179116	KT179156	KT179196
Solanum triflorum	KT178595	KT178629	KT178674	KT178718	KT178758	KT178798	KT178838	KT178878	KT178918	KT178958	KT178998	KT179038	KT179077	KT179117	KT179157	KT179197
Monarda fistulosa var mollis	KT178587	KT178630	KT178675	KT178719	KT178759	KT178799	KT178839	KT178879	KT178919	KT178959	KT178999	KT179039	KT179068	KT179118	KT179158	KT179198
Nepeta cataria	KT178589	KT178632	KT178677	KT178721	KT178761	KT178801	KT178841	KT178881	KT178921	KT178961	KT179001	KT179041	KT179069	KT179120	KT179160	KT179200
Salvia nemorosa	KT178588	KT178631	KT178676	KT178720	KT178760	KT178800	KT178840	KT178880	KT178920	KT178960	KT179000	KT179040	KT179070	KT179119	KT179159	KT179199
Teucrium canadense	KT178590	KT178637	KT178689	KT178725	KT178765	KT178805	KT178845	KT178885	KT178925	KT178965	KT179005	KT179045	KT179080	KT179124	KT179164	KT179204
Verbascum thapsus	KT178586	KT178635	KT178680	KT178724	KT178764	KT178804	KT178844	KT178884	KT178924	KT178964	KT179004	KT179044	KT179073	KT179123	KT179163	KT179203
Penstemon angustifolius	KT178584	KT178633	KT178678	KT178722	KT178762	KT178802	KT178842	KT178882	KT178922	KT178962	KT179002	KT179042	KT179071	KT179121	KT179161	KT179201
Penstemon gracilis	KT178585	KT178634	KT178679	KT178723	KT178763	KT178803	KT178843	KT178883	KT178923	KT178963	KT179003	KT179043	KT179072	KT179122	KT179162	KT179202
Verbena hastata	KT178592	KT178636	KT178681	KT178726	KT178766	KT178806	KT178846	KT178886	KT178926	KT178966	KT179006	KT179046	KT179081	KT179125	KT179165	KT179205
Veronica americana	KT178591	KT178638	KT178682	KT178727	KT178767	KT178807	KT178847	KT178887	KT178927	KT178967	KT179007	KT179047	KT179074	KT179126	KT179166	KT179206
Plantago patagonica	KT178598	KT178639	KT178693	KT178728	KT178768	KT178808	KT178848	KT178888	KT178928	KT178968	KT179008	KT179048	KT179088	KT179127	KT179167	KT179207
Impatiens capensis	KT178603	KT178643	KT178690	KT178732	KT178772	KT178812	KT178852	KT178892	KT178932	KT178972	KT179012	KT179052	KT179085	KT179131	KT179171	KT179211
Convolvulus arvensis	KT178580	KT178640	KT178686	KT178729	KT178769	KT178809	KT178849	KT178889	KT178929	KT178969	KT179009	KT179049	KT179092	KT179128	KT179168	KT179208
Ipomoea leptophylla	KT178581	KT178641	KT178687	KT178730	KT178770	KT178810	KT178850	KT178890	KT178930	KT178970	KT179010	KT179050	KT179093	KT179129	KT179169	KT179209
Evolvulus nuttallianus	KT178582	KT178642	KT178688	KT178731	KT178771	KT178811	KT178851	KT178891	KT178931	KT178971	KT179011	KT179051	KT179094	KT179130	KT179170	KT179210
Comandra umbellata	KT178604	KT178654	KT178692	KT178733	KT178773	KT178813	KT178853	KT178893	KT178933	KT178973	KT179013	KT179053	KT179090	KT179132	KT179172	KT179212
Galium aparine	KT178617	KT178653	KT178694	KT178734	KT178774	KT178814	KT178854	KT178894	KT178934	KT178974	KT179014	KT179054	KT179089	KT179133	KT179173	KT179213
Silene antirrhina	KT178605	KT178656	KT178695	KT178735	KT178775	KT178815	KT178855	KT178895	KT178935	KT178975	KT179015	KT179055	KT179086	KT179134	KT179174	KT179214
Silene vulgaris	KT178606	KT178657	KT178696	KT178736	KT178776	KT178816	KT178856	KT178896	KT178936	KT178976	KT179016	KT179056	KT179087	KT179135	KT179175	KT179215
Campanula rotundifolia	KT178607	KT178655	KT178697	KT178737	KT178777	KT178817	KT178857	KT178897	KT178937	KT178977	KT179017	--	KT179096	KT179136	KT179176	KT179216

Mitochondrial (mtDNA) Page 1 of 2:

**Species**	atp4	atp8	coxIII	nad3	nad4L	nad9
Achillea millefolium	KT179217	KT179280	KT179343	KT179406	KT179469	KT179532
Carduus nutans	KT179218	KT179281	KT179344	KT179407	KT179470	KT179533
Cirsium canescens	KT179219	KT179282	KT179345	KT179408	KT179471	KT179534
Cirsium undulatum	KT179220	KT179283	KT179346	KT179409	KT179472	KT179535
Erigeron bellidiastrum	KT179221	KT179284	KT179347	KT179410	KT179473	KT179536
Erigeron strigosus	KT179222	KT179285	KT179348	KT179411	KT179474	KT179537
Erigeron philadelphicus	KT179223	KT179286	KT179349	KT179412	KT179475	KT179538
Solidago gigantea	KT179224	KT179287	KT179350	KT179413	KT179476	KT179539
Heterotheca stenophylla var stenophylla	KT179225	KT179288	KT179351	KT179414	KT179477	KT179540
Heterotheca villosa	KT179226	KT179289	KT179352	KT179415	KT179478	KT179541
Tragopogon dubius	KT179227	KT179290	KT179353	KT179416	KT179479	KT179542
Senecio integerrimus	KT179228	KT179291	KT179354	KT179417	KT179480	KT179543
Grindelia squarrosa var squarrosa	KT179229	KT179292	KT179355	KT179418	KT179481	KT179544
Xanthisma spinulosum	KT179230	KT179293	KT179356	KT179419	KT179482	KT179545
Solidago missouriensis	KT179231	KT179294	KT179357	KT179420	KT179483	KT179546
Gutierrezia sarothrae	KT179232	KT179295	KT179358	KT179421	KT179484	KT179547
Lygodesmia juncea	KT179233	KT179296	KT179359	KT179422	KT179485	KT179548
Lactuca ludoviciana	KT179234	KT179297	KT179360	KT179423	KT179486	KT179549
Echinacea angustifolia	KT179235	KT179298	KT179361	KT179424	KT179487	KT179550
Helianthus pauciflorus	KT179236	KT179299	KT179362	KT179425	KT179488	KT179551
Helianthus petiolaris	KT179237	KT179300	KT179363	KT179426	KT179489	KT179552
Helianthus tuberosa	KT179238	KT179301	KT179364	KT179427	KT179490	KT179553
Ratibida columnifera	KT179239	KT179302	KT179365	KT179428	KT179491	KT179554
Silphium integrifolum	KT179240	KT179303	KT179366	KT179429	KT179492	KT179555
Liatris glabrata	KT179241	KT179304	KT179367	KT179430	KT179493	KT179556
Vernonia baldwinii	KT179242	KT179305	KT179368	KT179431	KT179494	KT179557
Rudbeckia hirta var pulcherrima	KT179243	KT179306	KT179369	KT179432	KT179495	KT179558
Antennaria neglecta	KT179244	KT179307	KT179370	KT179433	KT179496	KT179559
Heliopsis helianthoides var occidentalis	KT179245	KT179308	KT179371	KT179434	KT179497	KT179560
Symphoricarpus occidentalis	KT179246	KT179309	KT179372	KT179435	KT179498	KT179561
Campanula rotundifolia	KT179247	KT179310	KT179373	KT179436	KT179499	KT179562
Cicuta maculata	KT179248	KT179311	KT179374	KT179437	KT179500	KT179563
Conium maculatum	KT179249	KT179312	KT179375	KT179438	KT179501	KT179564

Mitochondrial (mtDNA) Page 2 of 2:

**Species**	atp4	atp8	coxIII	nad3	nad4L	nad9
Zizia aurea	KT179250	KT179313	KT179376	KT179439	KT179502	KT179565
Convolvulus arvensis	KT179251	KT179314	KT179377	KT179440	KT179506	KT179566
Ipomoea leptophylla	KT179252	KT179315	KT179378	KT179441	KT179507	KT179567
Evolvulus nuttallianus	KT179253	KT179316	KT179379	KT179442	KT179508	KT179568
Physalis heterophylla	KT179254	KT179317	KT179380	KT179443	KT179503	KT179569
Physalis virginiana	KT179255	KT179318	KT179381	KT179444	KT179504	KT179570
Solanum carolinense	KT179256	KT179319	KT179382	KT179445	KT179505	KT179571
Solanum rostratum	KT179257	KT179320	KT179383	KT179446	KT179509	KT179572
Solanum triflorum	KT179258	KT179321	KT179384	KT179447	KT179510	KT179573
Comandra umbellata	KT179259	KT179322	KT179385	KT179448	KT179511	KT179574
Silene antirrhina	KT179260	KT179323	KT179386	KT179449	KT179512	KT179575
Silene vulgaris	KT179261	KT179324	KT179387	KT179450	KT179513	KT179576
Thelesperma filifolium	KT179262	KT179325	KT179388	KT179451	KT179514	KT179577
Hymenopappus tenuifolius	KT179263	KT179326	KT179389	KT179452	KT179515	KT179578
Antennaria howellii subsp neodioica	KT179264	KT179327	KT179390	KT179453	KT179516	KT179579
Plantago patagonica	KT179265	KT179328	KT179391	KT179454	KT179517	KT179580
Ellisia nyctelea	KT179266	KT179329	KT179392	KT179455	KT179518	KT179581
Monarda fistulosa var mollis	KT179267	KT179330	KT179393	KT179456	KT179519	KT179582
Salvia nemorosa	KT179268	KT179331	KT179394	KT179457	KT179520	KT179583
Penstemon angustifolia	KT179269	KT179332	KT179395	KT179458	KT179522	KT179584
Penstemon gracilis	KT179270	KT179333	KT179396	KT179459	KT179523	KT179585
Verbascum thapsus	KT179271	KT179334	KT179397	KT179460	KT179524	KT179586
Verbena hastata	KT179272	KT179335	KT179398	KT179461	KT179525	KT179587
Veronica americana	KT179273	KT179336	KT179399	KT179462	KT179526	KT179588
Teucrium canadense	KT179274	KT179337	KT179400	KT179463	KT179527	KT179589
Nepeta cataria	KT179275	KT179338	KT179401	KT179464	KT179521	KT179590
Asclepias verticillata	KT179276	KT179339	KT179402	KT179465	KT179528	KT179591
Androsace occidentalis	KT179277	KT179340	KT179403	KT179466	KT179529	KT179592
Imaptiens capensis	KT179278	KT179341	KT179404	KT179467	KT179530	KT179593
Galium aparine	KT179279	KT179342	KT179405	KT179468	KT179531	KT179594

Nuclear (nrDNA) Page 1 of 2:

**Species**	5.8S	18S	26S
Achillea millefolium	KT179595	KT179658	KT179721
Carduus nutans	KT179596	KT179659	KT179722
Cirsium canescens	KT179597	KT179660	KT179723
Cirsium undulatum	KT179598	KT179661	KT179724
Tragopogon dubius	KT179599	KT179662	KT179725
Senecio integerrimus	KT179600	KT179663	KT179726
Erigeron bellidiastrum	KT179601	KT179664	KT179727
Erigeron strigosus	KT179602	KT179665	KT179728
Erigeron philadelphicus	KT179603	KT179666	KT179729
Grindelia squarrosa var squarrosa	KT179604	KT179667	KT179730
Xanthisma spinulosum	KT179605	KT179668	KT179731
Solidago gigantea	KT179606	KT179669	KT179732
Solidago missouriensis	KT179607	KT179670	KT179733
Gutierrezia sarothrae	KT179608	KT179671	KT179734
Heterotheca stenophylla var stenophylla	KT179609	KT179672	KT179735
Heterotheca villosa	KT179610	KT179673	KT179736
Lygodesmia juncea	KT179611	KT179674	KT179737
Lactuca ludoviciana	KT179612	KT179675	KT179738
Echinacea angustifolia	KT179613	KT179676	KT179739
Helianthus pauciflorus	KT179614	KT179677	KT179740
Helianthus petiolaris	KT179615	KT179678	KT179741
Helianthus tuberosa	KT179616	KT179679	KT179742
Thelesperma filifolium	KT179617	KT179680	KT179743
Ratibida columnifera	KT179618	KT179681	KT179744
Hymenopappus tenuifolius	KT179619	KT179682	KT179745
Silphium integrifolum	KT179620	KT179683	KT179746
Liatris glabrata	KT179621	KT179684	KT179747
Vernonia baldwinii	KT179622	KT179685	KT179748
Rudbeckia hirta var pulcherrima	KT179623	KT179686	KT179749
Antennaria howellii subsp neodioica	KT179624	KT179687	KT179750
Antennaria neglecta	KT179625	KT179688	KT179751
Heliopsis helianthoides var occidentalis	KT179626	KT179689	KT179752
Symphoricarpus occidentalis	KT179627	KT179690	KT179753

Nuclear (nrDNA) Page 2 of 2:

**Species**	5.8S	18S	26S
Campanula rotundifolia	KT179628	KT179691	KT179754
Cicuta maculata	KT179629	KT179692	KT179755
Conium maculatum	KT179630	KT179693	KT179756
Zizia aurea	KT179631	KT179694	KT179757
Convolvulus arvensis	KT179632	KT179695	KT179758
Ipomoea leptophylla	KT179633	KT179696	KT179759
Evolvulus nuttallianus	KT179634	KT179697	KT179760
Physalis heterophylla	KT179635	KT179698	KT179761
Physalis virginiana	KT179636	KT179699	KT179762
Solanum carolinense	KT179637	KT179700	KT179763
Solanum rostratum	KT179638	KT179701	KT179764
Solanum triflorum	KT179639	KT179702	KT179765
Ellisia nyctelea	KT179640	KT179703	KT179766
Monarda fistulosa var mollis	KT179641	KT179704	KT179767
Salvia nemorosa	KT179642	KT179705	KT179768
Nepeta cataria	KT179643	KT179706	KT179769
Penstemon angustifolia	KT179644	KT179707	KT179770
Penstemon gracilis	KT179645	KT179708	KT179771
Verbascum thapsus	KT179646	KT179709	KT179772
Verbena hastata	KT179647	KT179710	KT179773
Veronica americana	KT179648	KT179711	KT179774
Teucrium canadense	KT179649	KT179712	KT179775
Asclepias verticillata	KT179650	KT179713	KT179776
Androsace occidentalis	KT179651	KT179714	KT179777
Imaptiens capensis	KT179652	KT179715	KT179778
Plantago patagonica	KT179653	KT179716	KT179779
Galium aparine	KT179654	KT179717	KT179780
Silene antirrhina	KT179655	KT179718	KT179781
Silene vulgaris	KT179656	KT179719	KT179782
Comandra umbellata	KT179657	KT179720	KT179783
