# Supplementary figures and images for: Biodiversity assessment among two Nebraska prairies: a comparison between traditional and phylogenetic diversity indices
Source: Biodivers Data J. 2015 Jul 17;(3):e5403. doi: 10.3897/BDJ.3.e5403 (PMC4549632; doi:10.3897/BDJ.3.e5403)

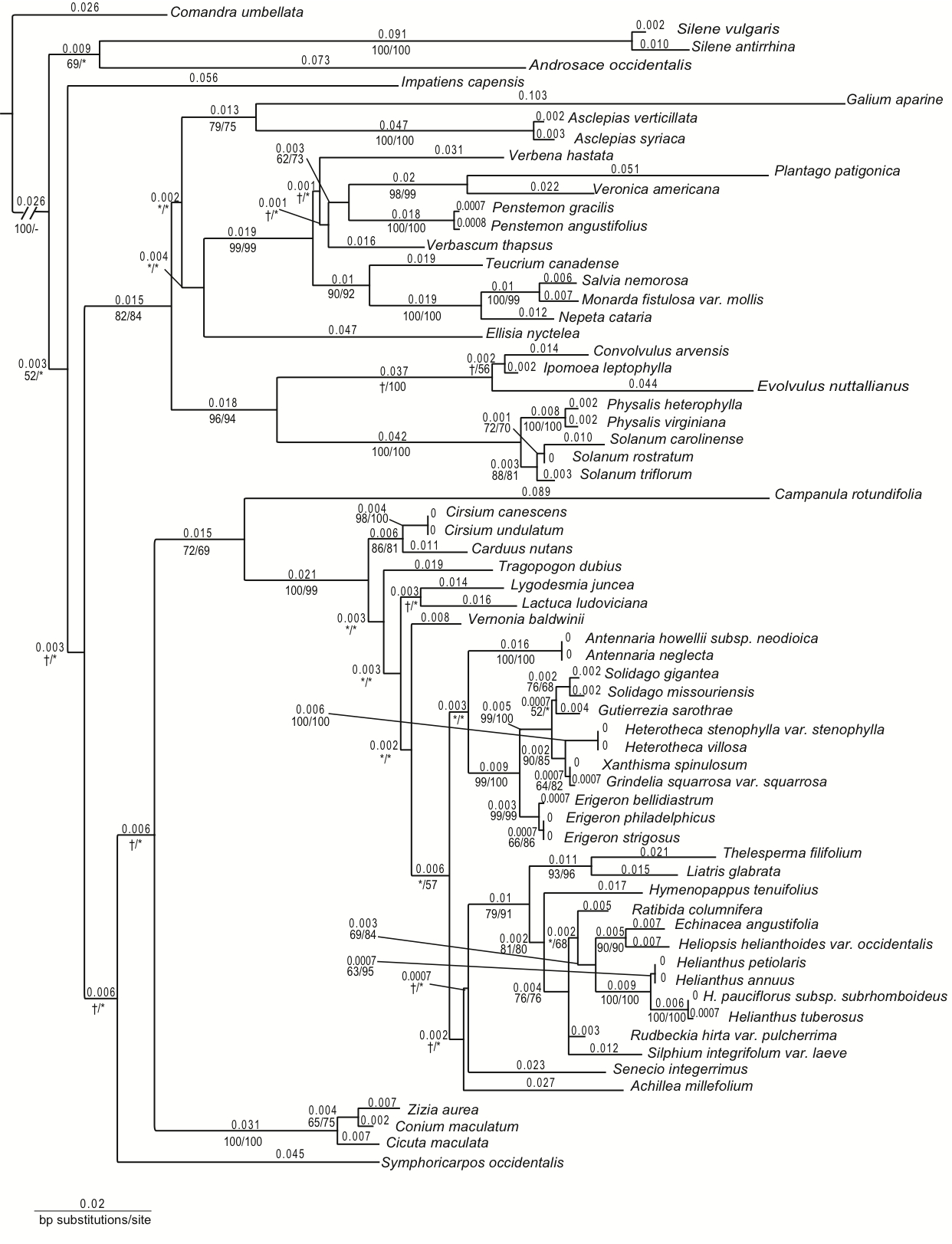

Supplement: Supplementary material 3 — rbcL Phylogeny [file biodiversity_data_journal-3-e5403-s003.jpg]

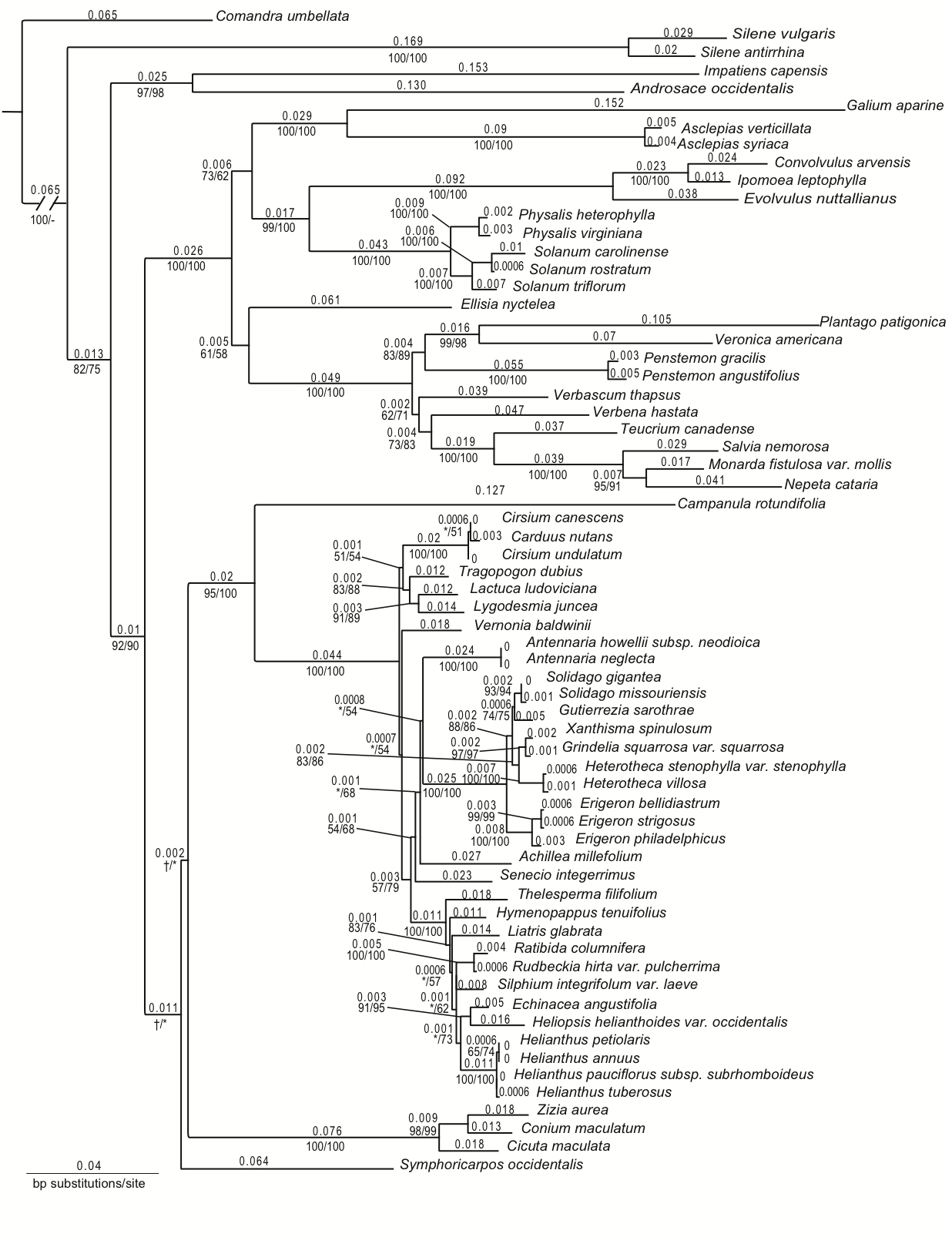

Supplement: Supplementary material 4 — matK Phylogeny [file biodiversity_data_journal-3-e5403-s004.jpg]

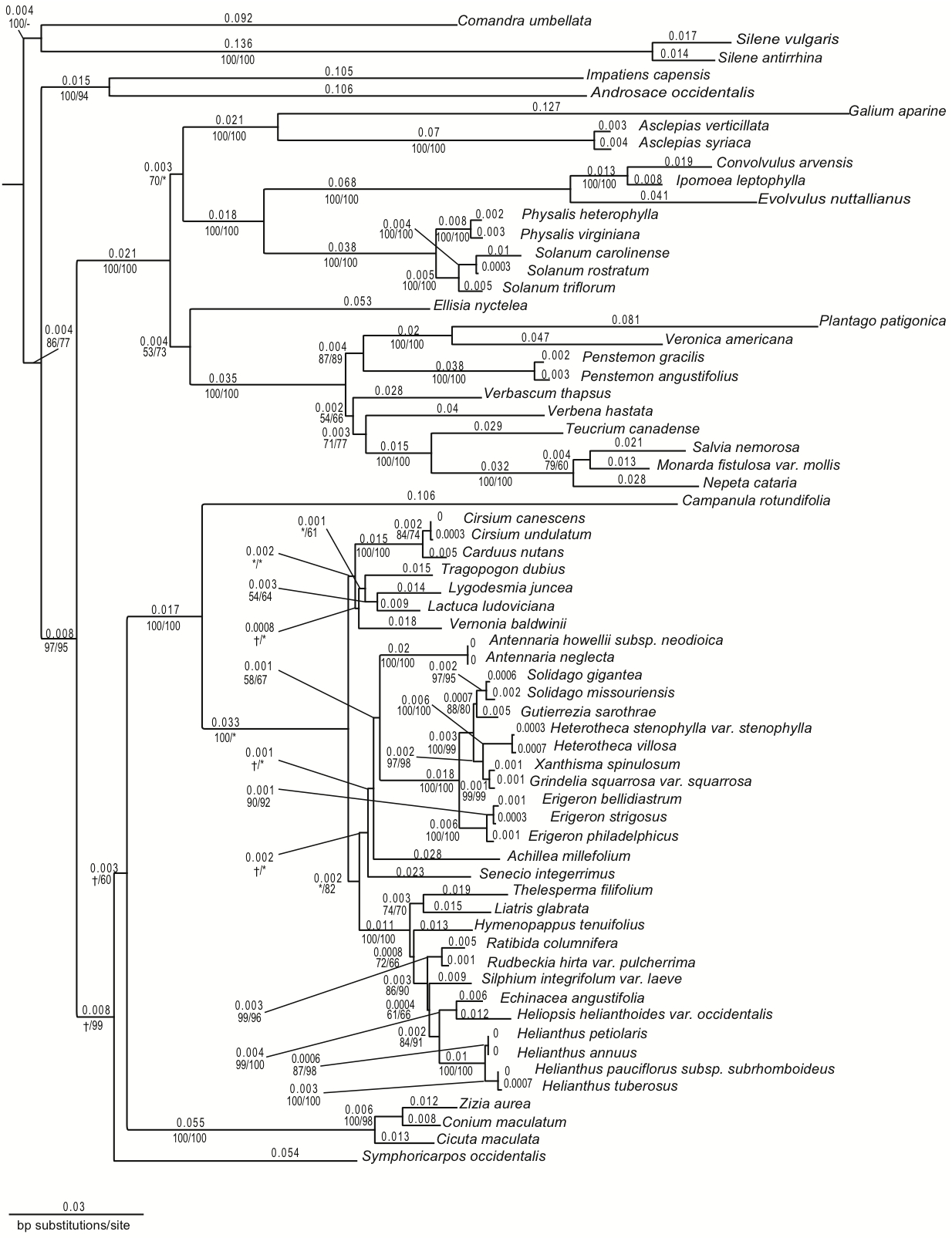

Supplement: Supplementary material 5 — rbcL + matK Phylogeny [file biodiversity_data_journal-3-e5403-s005.jpg]
